# Synergistic Spin-Mediated Catalysis for the Oxygen Evolution Reaction

**DOI:** 10.1021/jacs.5c14127

**Published:** 2025-11-10

**Authors:** Aravind Vadakkayil, Fiham Fahim, Wiley A. Dunlap-Shohl, Michael Vullo, Brian P. Bloom, David H. Waldeck

**Affiliations:** Chemistry Department, 6614 University of Pittsburgh, Pittsburgh, Pennsylvania 15260, United States

## Abstract

The oxygen evolution reaction (OER), the anodic half-reaction in water electrolysis, remains a critical bottleneck to the widespread adoption of a hydrogen-based economy. While spin polarization of reaction intermediates has been shown to enhance the performance of the OER, existing studies often overlook the distinctions between different ways of spin-polarizing intermediates and their correlation with catalytic efficiency. In this study, we conduct a comprehensive analysis of spin-mediated catalysis, revealing that the efficiency of the OER is highly sensitive to the nature and magnitude of the spin polarization applied. More significantly, we explore the interplay among different methods for generating spin polarization at catalysts and demonstrate how their combination can have a constructive or destructive impact on catalytic performance. To provide deeper insight into the mechanisms driving spin-controlled catalysis, we interpreted these findings using a statistical model.

## Graphical abstract

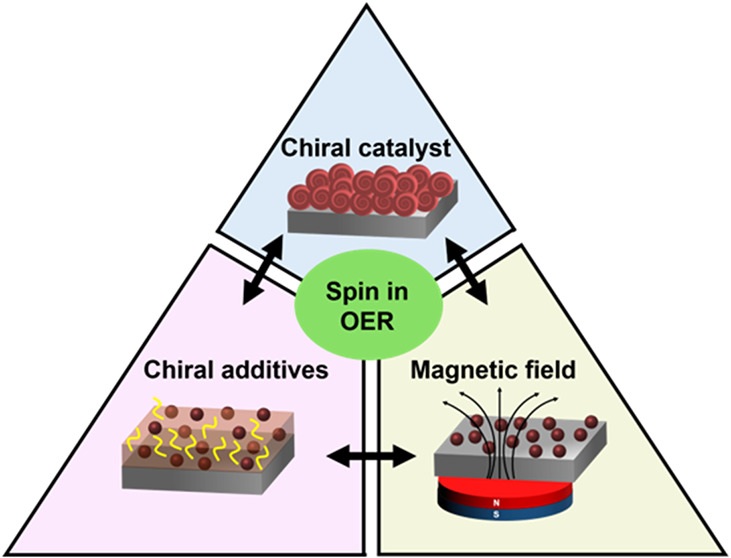

## Introduction

The global transition toward sustainable energy requires the development of efficient and scalable technologies for energy conversion and storage. Energy-related electrocatalytic reactions play a critical role in this regard,^1–3^ with electron spin polarization emerging as a powerful and accessible method to enhance catalytic efficiency, selectivity, and stability.^4–8^ Two primary mechanisms are used for implementing electron spin polarization during electrocatalysis: (1) by applying an external magnetic field to paramagnetic or ferromagnetic catalysts^9–12^ and (2) by leveraging the chiral induced spin selectivity (CISS) effect,^13–15^ the phenomenon by which charge displacement currents in chiral materials are accompanied by a spin polarization of the transmitted carriers.^5,16^ Spin-mediated catalysis has already been shown to affect electrochemical processes such as the oxygen evolution reaction (OER),^4,17–19^ oxygen reduction reaction,^20,21^ carbon dioxide reduction reaction,^22,23^ and nitrogen reduction reaction.^24,25^ In OER, the reaction product is ground-state oxygen, which exists in a triplet state (^3^O_2_, ^3^∑_g_^–^), while singlet oxygen (^1^O_2_, ^1^Δ_g_) lies at least ∼1 eV higher in energy.^9,26^ Because the reactant water is a singlet, spin constraints in the reaction mechanism’s elementary steps can significantly influence the kinetics of the OER.

In this work, we use the OER to examine how different methods for promoting spin polarization/spin orientation at a catalyst’s surface compare with each other and to assess any synergies between them. For example, when an external magnetic field is applied to a ferromagnetic or paramagnetic catalyst, the active sites on the catalyst are spin polarized along the applied field direction, resulting in the intrinsic generation of spin-polarized currents during electrocatalysis. We define this phenomenon as a “global spin bias” (GSB), i.e., a uniform bias that acts on the entirety of the material. When the strength of the external magnetic field is varied, so is the magnitude of the GSB (within the limits of the magnetic properties of the material). As such, researchers have shown systematic changes in the OER activity with applied magnetization; Figure 1a–d plots a magnetocurrent, defined as $\frac{J_{\mathrm{ON}}-J_{\mathrm{OFF}}}{J_{\mathrm{OFF}}}\times 100\%$ , where *J*_ON_ and *J*_OFF_ correspond to the current response with and without an applied magnetic field at a given potential, for CoO_*x*_,^27^ Ni foam,^28^ NiFe layered double hydroxides,^29^ and La_0.7_Sr_0.2_Ca_0.1_MnO_3_ ^30^ catalysts, respectively. For all four different catalysts, an increase in magnetocurrent is observed with increasing applied magnetic field strength, i.e., increasing GSB.

**Figure 1:**
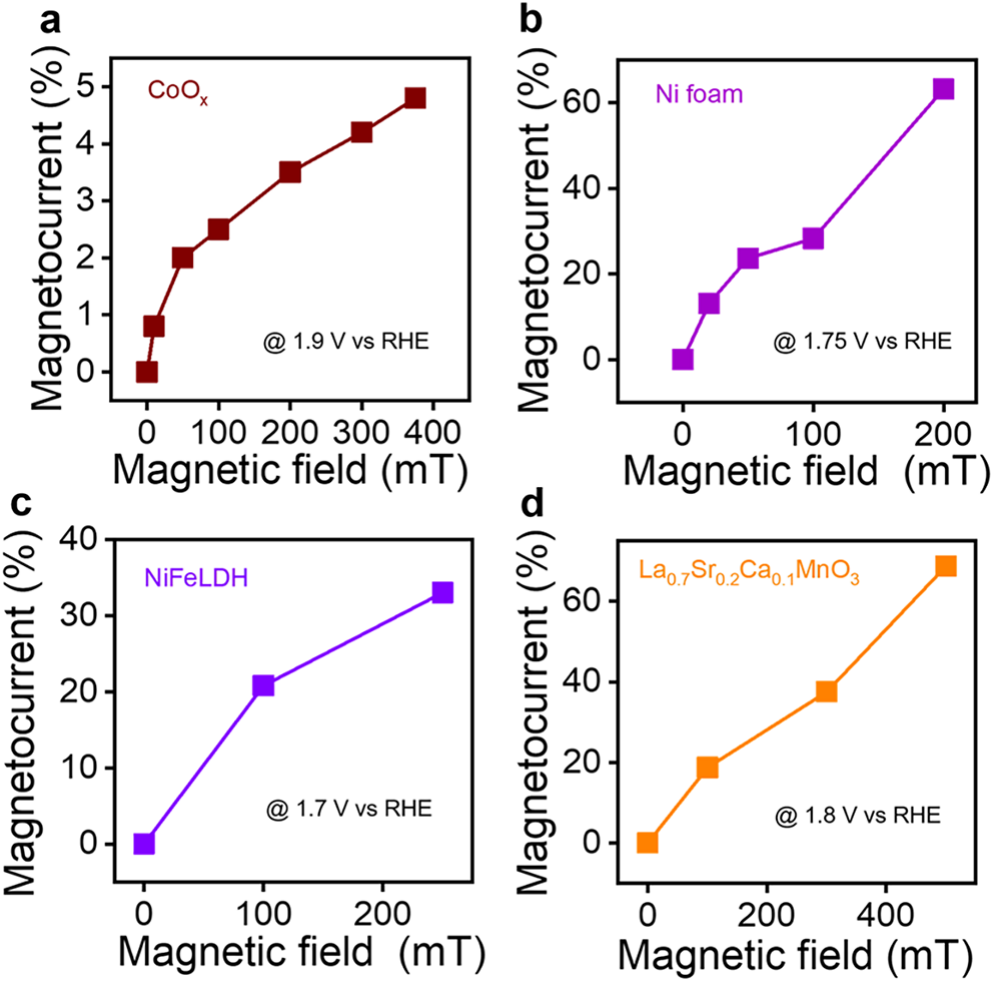
Effects of the global spin bias on the OER efficiency. Panels (a–d) summarize four literature works that show how an increase in magnetic field strength can increase the OER current, reported as a percentage increase on the magnetocurrent. These data are adapted from references 27–30 respectively.

A similar dependence on the GSB and the OER activity emerges via the CISS effect in chiral catalysts. As with magnetized ferromagnetic catalysts, the active sites of inherently chiral catalysts are spin oriented. Previous studies demonstrate that the magnitude of a material’s CISS-response correlates well with its chiroptical properties,^31–34^ implying that GSB should obey a similar relation. Indeed, recent studies have shown that, for the same metal oxide catalyst, an increase in circular dichroism (CD) correlates with a subsequent enhancement in OER activity.^35^ Figure 2 shows the percent improvement in OER current, CISS current, defined as $\frac{J_{\mathrm{Enantiopure}}-J_{\mathrm{Achiral}}}{J_{\mathrm{Achiral}}}\times 100\%$ , where *J*_Enantiopure_ and *J*_Achiral_ correspond to the current density from chiral and achiral catalysts, at a given potential, against the CD dissymmetry factor *g* for a CuO catalyst. Note that although the GSB created by a chiral catalyst is equivalent to that of a magnetic field in its effect on spin polarization, the underlying physical mechanisms are distinct, a fact that will become important when we consider the behavior of catalysts influenced by both.

**Figure 2:**
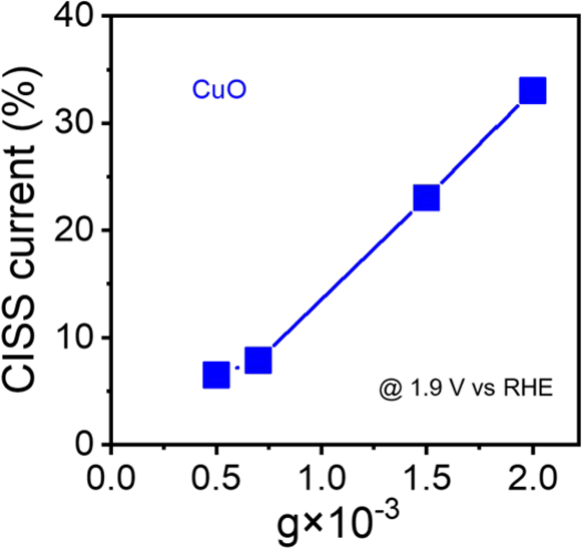
Effects of the global spin bias on the OER efficiency. The plot shows that an increase in circular dichroism of a chiral CuO catalyst correlates with an improved OER current for the catalyst. These data are adapted from ref 35.

Another method in which the CISS effect is used to spin polarize catalysts is through adjacent chiral electrolytes or molecular additives. For instance, chiral molecules added to a Nafion matrix supporting nanoparticle catalysts display improved OER activity compared to analogous racemic systems.^36^ Interestingly, the relationship between the chiral additive concentration and OER activity does not show a monotonic increase, particularly at higher concentrations; see Figure 4a, which replots the data from ref 36. In that work, the decrease in the level of the OER activity in the high-concentration regime was attributed to the random spatial orientation of chiral additives, which prevents uniform spin alignment of active sites. Because the chiral additive molecules are thought to bias the spin orientations of intermediates at localized sites, potentially differing in the preferred spin at different sites, we define this phenomenon as a “local spin bias” (LSB).

In this work, we develop a statistical model that correlates the OER activity with the strength of both a GSB and an LSB. The model incorporates features of the biases, including molecular properties of the additive, and predicts how different biases interact, revealing whether their interaction gives rise to constructive or destructive effects on catalytic performance. These predictions are compared against experiments to examine the model’s ability to describe these complex interactions. As depicted in Scheme 1, the model randomly assigns spatial distributions of reaction intermediates (step 1) and spin biases (step 2) on a square lattice. The active sites on patches of the catalyst have spin states assigned to the intermediates according to the strength of the prevailing spin bias at that site (step 3). The reaction products are calculated as either desired triplets (i.e., ^3^O_2_) or undesired singlets (e.g., H_2_O_2_ or ^1^O_2_), based on whether a given intermediate is aligned parallel or antiparallel to the nearest neighbor with which it “reacts” (step 4). By employing a Monte Carlo approach to construct many such lattices and calculating ensemble statistics on triplet and singlet products, we simulate the behavior of the catalyst and interrogate the effects of various spin biases on the OER efficiency.

**Scheme 1:**
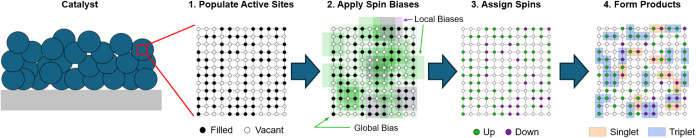
Calculation of Triplet and Singlet OER Products Using a Statistical Model^a^

a A patch of catalyst surface is modeled as a square lattice randomly occupied by spin-polarizable reaction intermediates (Step 1). This patch is subjected to a combination of global (i.e., from an intrinsically chiral catalyst or applied magnetic field; large green square) and/or local (i.e., from chiral additives in the vicinity of the catalyst surface; small green & purple squares) spin biases (Step 2). Lattice sites are randomly assigned to up or down spin states according to the net spin bias at each site (Step 3), and probabilistically paired with their nearest neighbors to form triplet products (blue) if their spins are aligned, or singlet products (orange) if they are opposed (Step 4). The principal figure of merit of each catalyst patch is the number of triplet products produced per 100 lattice sites. By repeating this process many times, the aggregate OER performance of an entire catalyst may be simulated as a response to the nature of the biases applied in Step 2 (e.g., their strengths and spatial distributions).

## Results and Discussion

### Global Spin Bias on OER

To model the effect of a GSB on the OER, we begin by representing a catalyst surface as a 20 × 20 square lattice randomly populated by spin-polarizable reaction intermediates, with a site occupation probability θ. (See Figures S1, S2, and S3, and the associated discussion for a step-by-step explanation of the model construction). Each occupied site is randomly assigned a spin-up or spin-down orientation, with a spin-up probability of 0.5 + *B*_GSB_, where *B*_GSB_ represents the global spin bias from either a magnetic field applied on a magnetic catalyst or an intrinsically chiral catalyst. That is, when *B*_GSB_ = +0.5 (−0.5), the reaction intermediates, if present, are uniformly spin-polarized upward (downward); when *B*_GSB_ = 0, the reaction intermediate has an equal probability of being spin-up or spin-down, corresponding to a case of no net spin polarization. The *B*_GSB_ can take a range of values from −0.5 to 0.5.

To simulate the influence of spin polarization on radical intermediate coupling, adjacent occupied sites are assumed to pair with their nearest neighbors, “reacting” with probability *P*_↑↓_ for spins aligned antiparallel (producing singlet byproducts like H_2_O_2_) and with probability *P*_↑↑_ for spins aligned parallel (producing the desired triplet product O_2_). After all of the sites are given the chance to react, we calculate the “triplet yield,” i.e., the number of triplet products formed per 100 lattice sites. In the simulations described below, we set *P*_↑↓_ = 0, i.e., assume that singlet formation is negligible at the high-pH and potential conditions under which our experiments are performed, consistent with previous measurements showing high Faradaic efficiency under basic conditions.^18,48^ Under these conditions, the triplet yield becomes analogous to the measured OER specific activity (SA), i.e., the current (proportional to the number of triplets formed) normalized to the electrochemically accessible surface area (ECSA; proportional to the number of active sites). Results for other values of *P*_↑↓_ and *P*_↑↑_ are described in Figure S4 (Supporting Information).

To explore the impact of model parameters (e.g., θ, *B*_GSB_, *P*_↑↓_, *P*_↑↑_) on the triplet yield, or triplets per 100 lattice sites, we average results over 1,000 randomly generated lattices per parameter set. Figure 3 shows a representative case: θ = 0.5, *P*_↑↑_ = 0.1, and *P*_↑↓_ = 0, with the variable *B*_GSB_. A representation of the spread in triplet yield across all of the simulated lattices for a given point is shown in Figure S5. The triplet yield follows a near-parabolic curve (*R* ^2^ > 0.9995) as a function of *B*_GSB_, with a minimum at *B*_GSB_ = 0 (no spin bias) and increasing yield as |*B*_GSB_| grows. This result implies that the formation of spin-aligned (triplet) O_2_ products should grow quadratically with spin polarization from a global spin bias on a catalyst surface. Note that when varying *P*_↑↑_ and *P*_↑↓_, the same parabolic trend persists; however, a higher *P*_↑↓_ reduces the triplet yield (see Figure S4). Although the triplet yield increases parabolically with GSB, the bias itself should be a saturating function of the physical influence, i.e., magnetic field or chirality. Assuming that the reaction intermediates behave in aggregate like a two-state paramagnet with individual magnetic moments *m* under applied field *H*, $B_{\mathrm{GSB}}=\frac{1}{2}\tan h\left(\frac{\mathrm{mH}}{k_{B}T}\right)$ −that is, the GSB is proportional to the magnetization of the ensemble, rather than the field that produces it; see Supporting Information for additional discussion.^37^ This interpretation accounts for the sublinear to linear dependences of magnetocurrent on applied field observed in Figure 1. (Also, note that since the triplet yield is dictated by the magnitude of this magnetization but insensitive to its direction, it increases regardless of the sign of the bias.) Collectively, the model predicts that any nonzero GSB enhances OER efficiency by increasing the availability of spin-aligned (triplet-forming) intermediate pairings on the catalyst surface.

**Figure 3:**
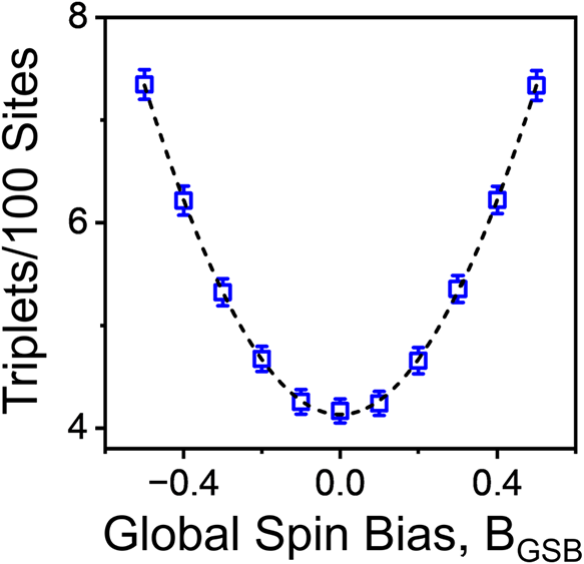
Results of Monte Carlo simulations of triplet yield of an OER catalyst in response to a changing chiral bias or applied magnetic field, i.e., *B*_GSB_, at a fixed reaction intermediate coverage parameter θ = 0.5 and triplet and singlet formation probability parameters *P*_↑↑_ = 0.1 and *P*_↑↓_ = 0. Each data point represents the mean of the outcomes from 1000 independent simulations corresponding to the same combination of parameters; error bars represent 95% confidence intervals for the mean. The dashed line is a parabolic fit of the simulation results.

### Local Spin Bias on OER

In previous work,^36^ we used camphorsulfonic acid (CSA) as a chiral additive to induce a local spin bias (LSB) and observed an increase in specific activity (SA) for the OER with enantiopure additives compared to racemic analogues; Figure 4a replots these data. To explain the effect of the chiral additive on the enhancement of the OER, we modified the Monte Carlo model described above to accommodate the effects of randomly distributed local spin biases. Briefly, the spin bias from chiral additives is assumed to act only locally on square subregions of the catalyst surface, termed their “domains of influence.” These domains are centered randomly on a secondary lattice overlaying the catalyst lattice and have a coverage governed by the additive occupation probability Θ (analogous to the experimentally controlled additive concentration).

**Figure 4:**
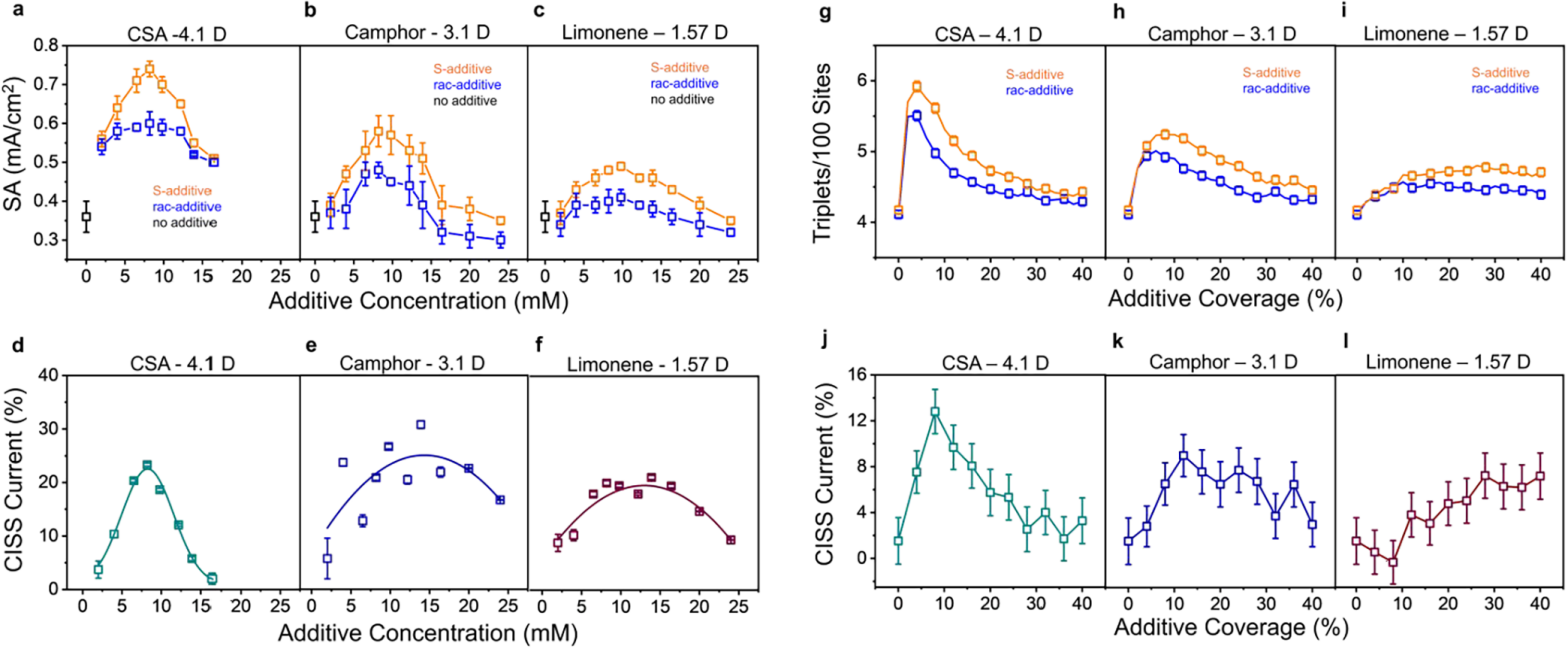
Experimental measurements of specific activity with chiral (orange) and racemic (blue) (a) CSA, (b) camphor, and (c) limonene additives, and corresponding CISS current (d) CSA (green), (e) camphor (dark blue), and (f) limonene (maroon) at an overpotential of 350 mV as a function of additive loading in the Fe_0.7_Co_2.3_O_4_ catalyst support. Each experimental data point represents data from three independently prepared electrodes, and the error bars are their standard deviation. Monte Carlo simulations of triplet yield (g–i) and CISS current (j–l) on catalyst surfaces in the presence of additives with (g, j) large, (h, k) intermediate, and (i, l) small domains of influence and spin biases, corresponding respectively to large, intermediate, and small electric dipole moments. In panels g–l, error bars represent 95% confidence intervals about the mean of 1000 simulations.

For convenience, we assume that the two lattices have identical geometry, and each additive molecule applies a local spin bias, *B*_LSB_ = *S*_A_|*B*_LSB_|, where |*B*_LSB_| is a constant model parameter and its sign *S*_A_ = ±1 represents the additive’s preference for spin-up or spin-down intermediates. The sign *S*_A_ is calculated as a product of two variables: the additive enantiomer *E* = ±1 according to whether it is S or R; and the additive orientation *A* = ±1 according to whether its electric dipole moment points upward or downward. Note that this choice accounts for the known dependence of CISS-based spin filtering on a chiral molecule’s dipole moment direction.^38^ Enantiomers are assigned according to a probability parameter ε, analogous to the enantiopurity of the additive in experiments: ε = 0.5 for a racemic mixture, and ε = 1 (or 0) for an enantiopure S (or R) population. Additive orientations are assigned up or down values by an analogous process governed by a probability parameter α, which we set to 0.8 in the simulations discussed below to account for the likely scenario of moderate orientational disorder. The net local spin bias *B*_LSB,net_ at a site falling within the domains of influence of *N* nearby additives is computed as

$$B_{\mathrm{LSB},\mathrm{net}}=\frac{1}{N}\sum_{k=1}^{N}S_{A,k}\left|B_{\mathrm{LSB}}\right|$$

This scheme sums the individual bias contributions and scales it to the range of −0.5 to 0.5. This bias combination penalizes cases of misaligned additives relatively harshly and reflects the capability of heterochiral systems to dramatically inhibit the potency of the CISS effect.^39^ Despite the more complex spatial distribution of LSBs relative to a global bias, the parabolic relationship between the bias and triplet yield is maintained, as shown in Figure S10.

This model provides a rationale for the enhancement of SA by enantiopure (e.g., S-CSA) over racemic (e.g., rac-CSA) additives and the general evolution of specific activity with additive loading as reflected by Figure 4a. Note that the same outcome is reproduced when R-CSA is used in place of S-CSA (Figure S6). At low loading, SA is enhanced due to increased triplet production in regions of the catalyst affected by the local bias of the additives. Since this enhancement occurs regardless of the sign of the LSB, both S- and rac-CSA initially lead to comparable improvements over the additive-free catalyst. As the additive loading continues to increase, however, the SA of the enantiopure additive continues to improve over that of the racemate, which we attribute to destructive interference between oppositely aligned LSBs of overlapping additives. This effect is more severe for racemic than enantiopure additives, because the clash between LSBs arises from the random distribution of enantiomers as well as spatial misorientation. As the additive loading increases further, SA declines regardless of additive enantiopurity because the probability of clashing LSB from misalignment increases with their density. This rise-and-fall behavior may thus be summarized as a combination of two basic effects: SA increases with additive coverage initially because more of the catalyst’s surface is affected by a spin bias; but once additive overlap becomes significant, their spin biases may oppose one another, causing SA to decline.

While our previous modeling work reproduced this peaking behavior,^36^ it did not yield the experimentally observed convergence between the SAs of S-CSA and rac-CSA catalysts at high coverage, suggesting that an additional physical process was not captured by this modeling. We hypothesized that the CISS effect regulating the LSB from the additives should be affected by their electric dipole moment, μ. Thus, we measured the SA of catalysts prepared using camphor (|μ| = 3.1 D) and limonene (|μ| = 1.57 D) additives (whose dipole moments were obtained from literature),^40^ which both possess a weaker |μ| than CSA (|μ| = 4.1 D, whose dipole moment was calculated as described in the Methods section). Note that the chiral additives do not adsorb onto the catalyst. This conclusion is supported by the ECSAs of catalyst films prepared with and without additives being approximately the same (Figure S7 and Table S1) and by CD measurements of S additives in Nafion catalyst ink suspensions, for which no chiroptical signatures from the catalyst are observed (Figure S8). Control experiments confirm that the observed current enhancement in the presence of chiral additives does not stem from additive oxidation; see Supporting Information in Figure S9. Figure 4a–c illustrates the specific activity at a 350 mV overpotential for an Fe_0.7_Co_2.3_O_4_ catalyst in 1 M NaOH with CSA, camphor, and limonene additives, respectively. Qualitatively, the SA change with additive loading behaves similarly for all three S- and racemic additives; i.e., they increase from a low value, reach a maximum, and then decrease. We observe that the CISS current also displays a similar rise-and-fall behavior (Figure 4d–f), reinforcing the finding that the advantages of enantiopure additives are realized at an intermediate value of the additive concentration. However, we note several important quantitative differences. First, the maximum specific activity of both S and racemic systems increases with |μ|, in agreement with the expectation of a stronger spin bias. Second, the maximum SA and CISS currents tend to be realized at smaller values of additive loading as |μ| increases. Finally, the convergence in SA between S and racemic systems at high additive loading is more pronounced when |μ| is large. These trends imply that the chiral additive |μ| is a crucial attribute of this catalyst scheme.

To probe the influence of |μ| in the context of the statistical model, we formalized its role in terms of three distinct effects: we assume that (i) the additive LSB strength |*B*_LSB_| scales monotonically with its |μ| (assigning values of 0.3, 0.4, and 0.5 to limonene, camphor, and CSA, respectively); (ii) the additive domain of influence also increases with |μ| (assigning 3 × 3, 5 × 5, and 7 × 7 squares to the above additives); and (iii) the electrostatic dipole–dipole interaction between nearby additives affects their orientation. The interaction energy *U*_DD_ scales with |μ|^2^:

$$U_{\mathrm{DD}}=\pm \frac{|{\mu |}^{2}}{4\pi {\epsilon_{r}\epsilon }_{0}r^{3}}$$

where ϵ_r_ is the dielectric constant of the intervening medium and ϵ_0_ is the permittivity of free space, *r* is the distance between the dipoles, and the sign is positive if the dipoles are parallel and negative if they are antiparallel. To incorporate these effects into the model, when assigning additive orientations *A*, we calculate an organization energy implied by the alignment parameter α, and determine the most energetically favorable state for each additive subject to the joint effects of this energy, along with the dipole–dipole interaction energy summed over neighboring additives. We then use a variation of the Metropolis algorithm^37^ to randomly flip each additive according to Boltzmann statistics until a quasi-equilibrium distribution of additive orientations is reached. Further details on this calculation are provided in the Supporting Information, and sample outcomes of LSB distributions are illustrated graphically in Figure S11.

Besides |μ|, this calculation entails three additional parameters: temperature *T*, lattice site spacing *d*, and dielectric constant ϵ_r_, which we take to be 300 K, 0.5 nm, and 4, respectively, for all simulations discussed below. (To retain the computational advantages of identical lattices for intermediates and additives, we set the site spacing to *d* = 5 Å, a compromise between the ∼3–4 Å active site distances in oxides^41–43^ and the ∼6–7 Å intermolecular spacing of chiral additives such as camphor and CSA, inferred from crystal structures.^44,45^ ) Otherwise, we set the reaction intermediate site occupancy to θ = 0.5, the triplet formation probability *P*_↑↑_ = 0.1, singlet formation probability *P*_↑↓_ = 0, ε = 0.5 for racemic additives, and 1 for S additives; and we allow the additive coverage parameter Θ to vary from 0 to 40%. The additive alignment factor, α, is set to 0.8 to ensure that the orientation energy exceeds *k*_B_ *T* (overcoming thermal disorder) yet remains small enough for dipole–dipole interactions to influence results. Smaller values (α = 0.5) would show no distinction between S and racemic additives, while larger values (α = 1) would prevent the yield curves from converging.

The results of these simulations for additives of different dipole moments are presented in Figures 4g–i,j–l, depicting the triplet yield of catalysts with enantiopure additives and the CISS current (i.e., the enhancement of triplet yield calculated as a difference between S and racemic catalysts over racemic alone), respectively. As in the experimental data, the maximum triplet yield increases with |μ| via the corresponding increase in |*B*_LSB_|. Furthermore, the peaks in triplet yield and CISS current are reached for lower values of Θ, and the rises are sharper, attributed to the increased domain area that requires fewer additives to completely cover the catalyst surface with their biases. Lastly, the decline of triplet yield and CISS current with additive coverage at high loadings becomes steeper as |μ| increases, due to the orientational dipole–dipole interactions promoting antiparallel alignments. Similar reasoning has been invoked to explain antiparallel ordering of dipoles in polar liquids such as DMSO and NMP.^46,47^ Additional simulations clarifying the importance of dipole–dipole interactions are provided in the Supporting Information and Figure S12. In summary, this model provides support for a mechanistic connection between the strength of a chiral additive’s |μ|, CISS, and catalyst activity. Note, however, that the model values are chosen to capture system trends rather than strict equivalence with specific additives.

### Combinatorial Effects of GSB and LSB on OER

Here, we investigate three combinations of the different spin biases, i.e., (i) chiral catalysts (*B*_GSB,C_) with chiral additives (*B*_LSB_), (ii) magnetized catalysts (*B*_GSB,M_) with chiral additives (*B*_LSB_), and (iii) chiral catalysts (*B*_GSB,C_) that are magnetized (*B*_GSB,M_). Because global spin biases from chiral catalysts and magnetic fields have different physical origins, we construct separate combination rules for their joint effects.

*Case (i)*: To incorporate the combined effects of a chiral GSB and LSB in our statistical model, we merge the approaches from parts 1 and 2, using a similar scheme to handle the combination of global and local biases. At each lattice site, the net bias is calculated as a weighted sum of the global spin bias and the net local spin bias from the additives:

$$B_{\mathrm{net}}=\{\begin{array}{l} \rho B_{\mathrm{GSB}}+\left(1-\rho \right)B_{\mathrm{LSB},\mathrm{net}},,N>0\\ B_{\mathrm{GSB}},,N=0 \end{array}$$

Here, *N* is the number of additives influencing the site, and 0 ≤ ρ ≤ 1 controls the relative influence of global bias versus local bias. High ρ values imply a dominant GSB, while ρ = 0 means an LSB dominates. Importantly, even when ρ = 0, the GSB still acts on additive-free regions, allowing complex mixed behaviors to emerge. We consider two basic scenarios for the two different biases: aligned (i.e., same spin direction) and opposed (i.e., opposite spin direction). In a representative simulation, we use the following parameters characteristic of CSA: reaction intermediate site occupancy θ = 0.5, reaction product formation parameters *P*_↑↓_ = 0, *P*_↑↑_= 0.1, additive chiral bias magnitude |*B*_LSB_| = 0.5, enantiopurity factor ε = 1, additive alignment factor α = 0.8, domain sizes 7 × 7, dipole moment μ = 4.1 D, temperature *T* = 300 K, lattice site spacing *d* = 0.5 nm, and dielectric constant ϵ_r_ = 4. We set ρ = 0.5, weighting the local additive bias *B*_LSB_ and global catalyst bias *B*_GSB_ equally. We then vary the *B*_GSB_ from −0.5 to 0.5 (negative for opposed biases, positive for aligned) and examine how the triplet yield depends on additive coverage Θ.

Figure 5a shows the case of an aligned GSB and LSB. When GSB is relatively weak (|*B*_GSB_ | ≤ 0.3), the triplet yield initially increases with additive concentration but further increases cause it to plateau and decline as overlapping additive domains begin to disrupt spin polarization. When the GSB is strong (*B*_GSB_ > 0.3), however, additives become consistently detrimental, with performance decreasing as the additive concentration increases. Figure 5b presents the opposed bias case, where a sharper decline in triplet yield is seen at large additive concentration due to destructive interference between the LSB and the GSB. Interestingly, at low additive loading (Θ < 1%) and weak GSB (*B*_GSB_ = −0.1), a synergistic regime manifests—triplet yields exceed those from either bias alone, despite their opposing directions. This synergistic regime arises because, when the additive population is relatively sparse, the improvement from the background GSB in the additive-free region of the catalyst outweighs the reduction in the effectiveness of the additives when they clash with the GSB. From this analysis, a simple design rule emerges: *chiral additives* (*B*_LSB_) *can improve performance when a global chiral bias B*_GSB_ *is weak but hinder performance when B*_GSB_ *is strong*.

**Figure 5:**
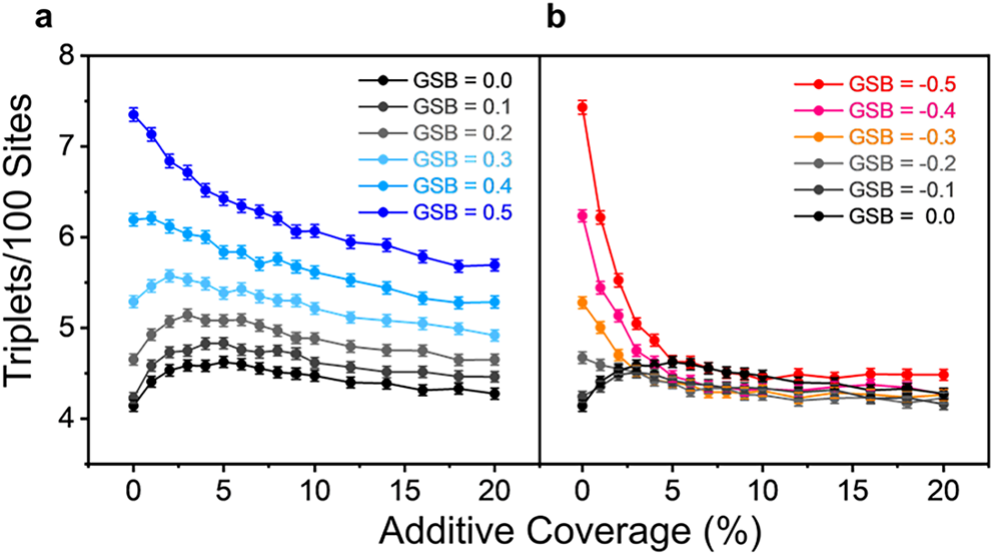
Monte Carlo simulations of the triplet yield of a catalyst experiencing a global spin bias from its intrinsic chirality, either aligned with (a) or opposed to (b) a net local spin bias due to the proximity of chiral additives as a function of additive loading. Each data point represents the mean of 1000 simulations, and the error bars represent 95% confidence intervals about this mean.

These conclusions are supported by experimental OER results on chiral D-Co_3_O_4_ catalysts with chiral CSA additives. Figure 6a presents the SA, measured at a 350 mV overpotential, for D-Co_3_O_4_ (violet, *B*_GSB,C_), D-Co_3_O_4_ + S-CSA (green, *B*_GSB,C_ + *B*_LSB_ aligned), and D-Co_3_O_4_ + R-CSA (brown, *B*_GSB,C_+ *B*_LSB_ opposed). In addition, rac-Co_3_O_4_ (black, no spin bias) and rac-Co_3_O_4_ + S-CSA (orange, *B*_LSB_) are plotted for comparison. The SA data are derived from linear sweep voltammograms shown in Figure S13; further details regarding the synthesis and characterization are provided in the Supporting Information. When the inherently chiral catalyst (*B*_GSB,C_) is paired with chiral additives (*B*_LSB_) having the same sign of spin bias (green symbol), a superior SA is observed over the inherently chiral catalyst (violet symbol) or achiral catalyst with chiral additives (orange) alone. Conversely, when *B*_GSB,C_ and *B*_LSB_ are opposed (brown symbol), destructive interference occurs, and the SA is less than that of *B*_LSB_ or *B*_GSB,C_ alone. This trend in catalyst activity, i.e., GSB and LSB act cooperatively when aligned and detrimentally when opposed, emerges naturally from the model under conditions in which the spin biases are approximately equal in strength. Figure 6b shows model simulations derived from the data in Figure 5, when *B*_GSB,C_ = ±0.2 and the |*B*_LSB_| = 0.5 with an additive coverage parameter Θ = 5%, i.e., the optimal value for maximizing triplet yield. The calculated trend in triplet yield matches the observed SA trend (i.e., aligned biases > local spin bias ≈ global spin bias > opposed biases > completely achiral system), illustrating how the simulated effects on spin polarization account for the variation in OER activity. Complementary measurements and discussion on L-Co_3_O_4_ are provided in the Supporting Information (Figure S14).

**Figure 6:**
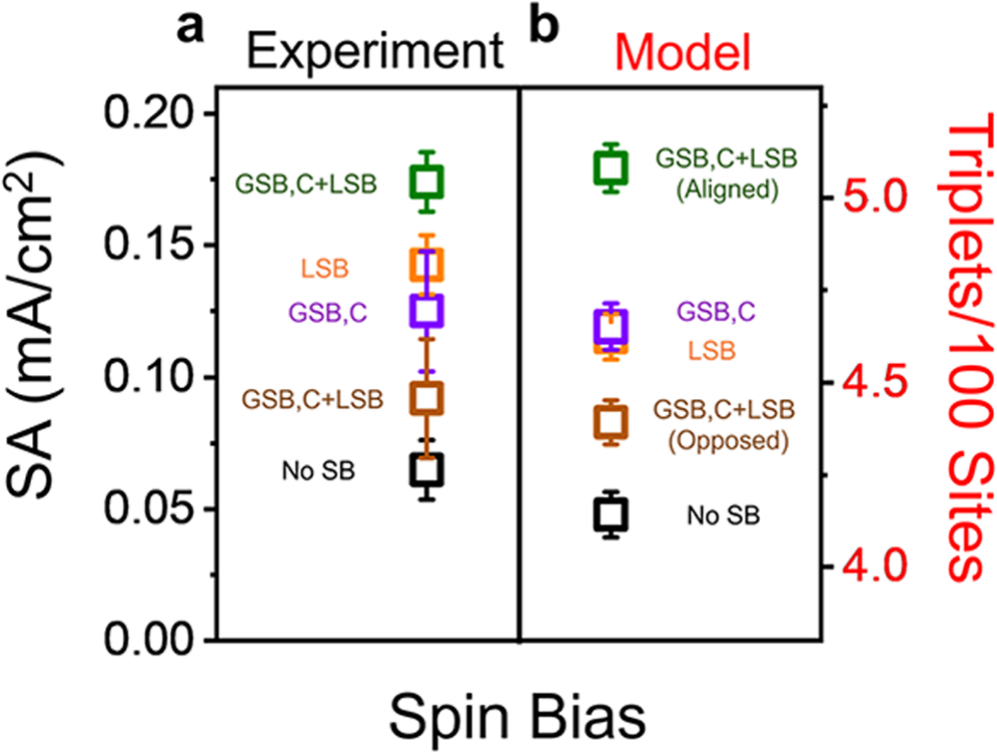
Measurements and simulations for the combination of the global spin bias from chirality with that of a local spin bias from additives. The experimental data (a) plots the SA at a 350 mV overpotential for chiral catalysts with the influence of an LSB. Measurements are shown for D-Co_3_O_4_ (violet), D-Co_3_O_4_ + S-CSA (green), D-Co_3_O_4_ + R-CSA (brown), and the black and orange symbols correspond to rac-Co_3_O_4_, rac-Co_3_O_4_ + R-CSA, respectively. Each experimental data point represents measurements from three independently prepared electrodes, and the error bars indicate the standard deviation. Monte Carlo simulations (b) of triplet yield using parameter combinations that reproduce the ordering of OER efficiency metrics, treating the effect of D-Co_3_O_4_ as a moderately weak global spin bias, *B*_GSB,C_ = ±0.2. The color coding of the symbols matches that of the corresponding cases for the experimental data. Each data point in the model represents the mean of 1000 simulations, and the error bars represent 95% confidence intervals for this mean.

*Case (ii)*: For the case in which the spin bias from an inherently chiral catalyst (*B*_GSB,C_) is replaced with that of a magnetized catalyst (*B*_GSB,M_), we use a somewhat different method to account for the different physical origins of the GSB. Instead of directly summing and rescaling the biases as above, we resolve the chiral bias into an effective magnetic field, sum it with the applied field, and recalculate the resulting net bias, yielding the following relation

$$B_{\mathrm{net}}=\frac{B_{\mathrm{LSB},\mathrm{net}}+B_{\mathrm{GSB},M}}{4B_{\mathrm{LSB},\mathrm{net}}{\cdot B}_{\mathrm{GSB},M}+1}$$

Details of the derivation of this expression are provided in the Supporting Information, and model predictions are displayed in Figure S15. To create Co_3_O_4_ catalysts that respond to magnetic fields, a pretreatment was performed, following a previously reported procedure;^48^ see Supporting Information and Figure S16 for further details on this process. Figure 7a presents experimental measurements of SA, at an overpotential of 350 mV, for rac-Co_3_O_4_ in the presence of a North magnetic field (brown), rac-Co_3_O_4_ with S-CSA and a South magnetic field (green), and rac-Co_3_O_4_ with a North magnetic field (purple). For comparison, rac-Co_3_O_4_ with S-CSA chiral additives and no magnetic field is also plotted (orange). The experimental SA data were obtained from LSVs presented in Figure S17. In these studies, the effect of chiral additives (*B*_LSB_, orange) gives rise to better SA than that for experiments with just a magnetized catalyst (*B*_GSB,M_, brown); i.e., the LSB is stronger than the GSB. Moreover, regardless of the alignment of the GSB relative to the LSB, the combination of *B*_GSB,M_ and *B*_LSB_ leads to improved SA; although aligned *B*_GSB,M_ + *B*_LSB_ (green) is still better than that of opposed *B*_GSB,M_ + *B*_LSB_ (violet). These trends in SA are captured by the model in the scenario of *B*_GSB,M_ = ±0.2 to represent the magnetic field (*B*_GSB,M_) with no intrinsic catalyst chirality; note that the additive coverage is 4%, i.e., the approximately optimal value with no applied field (Figure 7b), and ρ = 0 to reflect that additives provide the only chiral bias. Otherwise, the same parameters used in the simulations in Figure 6 are used here. Analogous experimental SA measurements of rac-Co_3_O_4_ with R-CSA uphold these trends and are presented in Figure S18.

**Figure 7:**
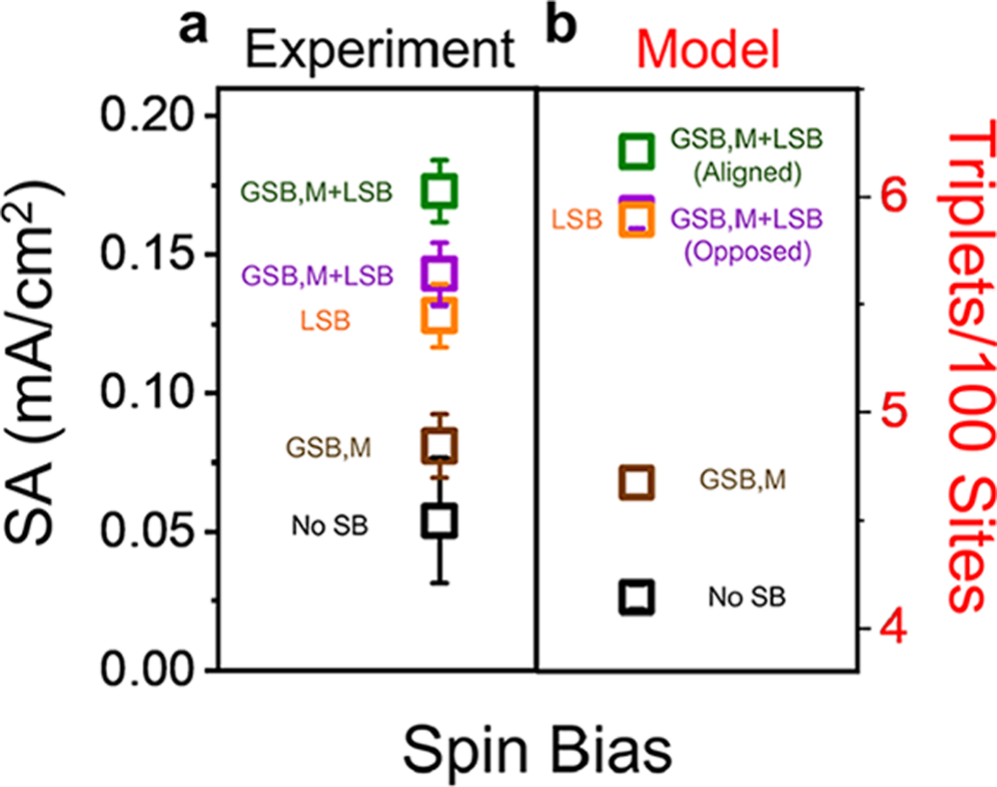
Measurements and simulations for the combination of a global spin bias from magnetization with a local spin bias from additives. The experimental data (a) plots the SA at a 350 mV overpotential for rac-Co_3_O_4_ combined with S-CSA and a South (North) magnetic field, which are represented in green (purple). In addition, data are shown for rac-Co_3_O_4_ without (black) and in the presence of a North magnetic field (brown), and rac-Co_3_O_4_ with S-CSA chiral additives and no field (orange). Each experimental data point represents measurements from three independently prepared electrodes, and the error bars indicate their standard deviation. Monte Carlo simulations (b) of triplet yield use parameter combinations that reproduce the ordering of OER efficiency metrics for that of an applied magnetic field as a weak global spin bias that is locally overridden by the spin bias of any additives present. The color coding of the symbols matches the corresponding cases for the experimental data. Each data point in the model represents the mean of 1000 simulations, and the error bars represent 95% confidence intervals about this mean; however, these may appear visually compressed due to the larger size of the data markers.

*Case (iii)*: Lastly, we consider the interplay between a chiral catalyst and an applied external magnetic field. Previous studies have demonstrated that coupling CISS with an external magnetic field can synergistically improve OER performance.^49,50^ Figure 8a shows the SA of D-Co_3_O_4_ with a South applied magnetic field (blue, *B*_GSB,C_ + *B*_GSB,M_) and D-Co_3_O_4_ with a North applied magnetic field (red, *B*_GSB,C_ + *B*_GSB,M_). In addition, D-Co_3_O_4_ with no field (violet, *B*_GSB,C_) and rac-Co_3_O_4_ in the presence (brown, *B*_GSB,M_) and absence of a magnetic field (black, No SB) are also shown. Applying a magnetic field to a chiral catalyst enhances the SA, with a South magnetic field (blue) giving rise to a larger enhancement than with a North magnetic field for D-Co_3_O_4_ (red symbol) over the case of no applied field (violet symbol). Such behavior implies that regardless of alignment, *B*_GSB,M_ and *B*_GSB,C_ reinforce one another, leading to a larger net spin bias. The LSVs used to derive the experimental SA data are shown in Figure S16, and complementary measurements performed on L-Co_3_O_4_ are presented in Figure S19.

**Figure 8:**
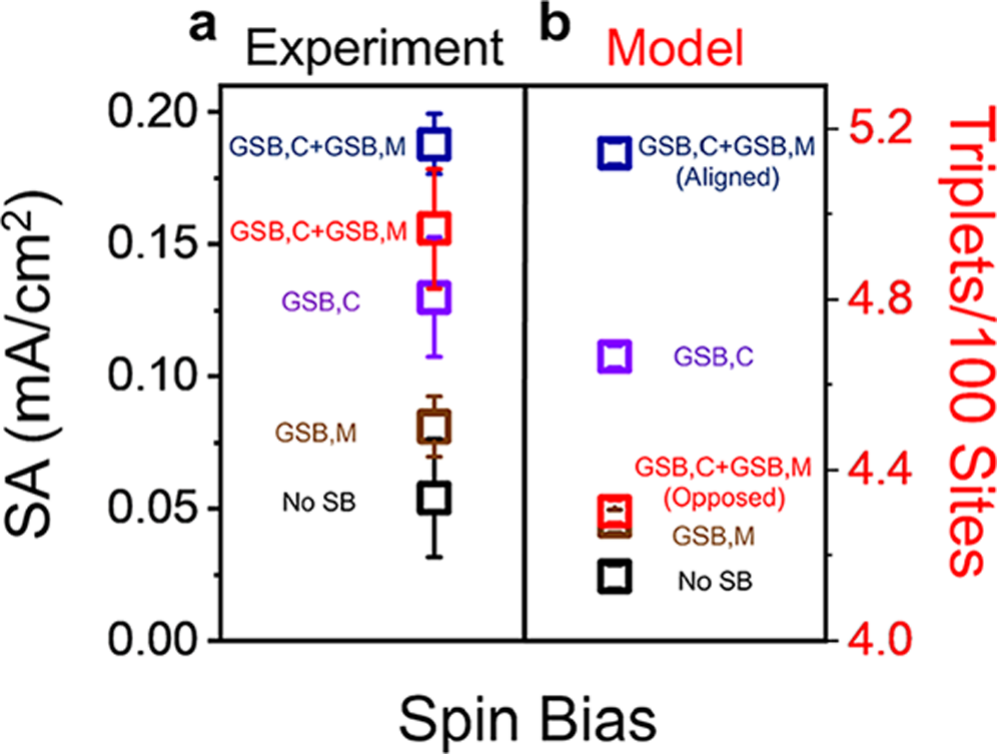
Measurements and simulations for the combination of the global spin bias from chirality with that of a global spin bias from magnetization. The experimental data (a) plots SA at a 350 mV overpotential for D-Co_3_O_4_ (violet) and D-Co_3_O_4_ with a North (red) and South (blue) applied magnetic field. The brown and black symbols show additional control measurements for the SA of rac-Co_3_O_4_ in the presence and absence of a North applied magnetic field, respectively. Each experimental data point represents measurements from three independently prepared electrodes, and the error bars indicate their standard deviation. Monte Carlo simulations (b) of triplet yield were performed using parameter combinations representing *B*_GSB,M_, *B*_GSB,C_, and their combinations. The color coding of the symbols matches the corresponding cases for the experimental data. Each data point in the model represents the mean of 1000 simulations, and the error bars represent 95% confidence intervals about this mean; however, these may appear visually compressed due to the larger size of the data markers.

To construct a net spin bias *B*_GSB,net_ for the combination of a magnetic field (*B*_GSB,M_) and an inherently chiral catalyst (*B*_GSB,C_), we employ the same combination method as above, replacing the net LSB with the GSB from the chiral catalyst. Since the chiral catalyst provides roughly twice the enhancement of the magnetic field, we represent the global bias from the former as *B*_GSB,C_ = 0.2 and that from the latter as *B*_GSB,M_ = ±0.1. According to the magnetic bias combination scheme, *B*_GSB,net_ ≈ +0.28 when the field orientation is aligned with the catalyst bias and *B*_GSB,net_ ≈ +0.11 when they are opposed. Using triplet yields drawn from the parabolic fit in Figure 3, we interpolate the net biases for these configurations in Figure 8b. While the model agrees with the experiments in that aligned *B*_GSB,M_ and *B*_GSB,C_ lead to improved SA over either *B*_GSB,M_ or *B*_GSB,C_, it fails to describe the improvement witnessed, when they are opposed. Notably, the system behaves qualitatively like an achiral catalyst with chiral additives and a magnetic field, suggesting that the chiral catalysts used for the experiments in Figure 8a do not display a uniform global bias. In particular, their spin bias may vary spatially, perhaps from inhomogeneous ligand-binding geometries during formation, with heterogeneity on scales larger than those of LSBs from additives. In this picture, an opposed magnetic field can still enhance the OER: achiral or weakly chiral regions benefit from the field regardless of orientation, while stronger chiral regions are reinforced or suppressed depending on alignment. Other synergistic effects may also play a role, such as chirality increasing the effective magnetic susceptibility of intermediates, making them more sensitive to spin polarization from the applied field. Indeed, CISS has been shown to influence the magnetic properties of materials.^51–53^

Future refinements of the model could improve quantitative agreement with experiment by explicitly modeling the temporal evolution of adsorbed intermediates and products so that specific activity emerges from adsorption, desorption, and reaction kinetics on heterogeneous crystal faces. More sophisticated treatments might also incorporate alternate pathways for adsorbate evolution, lattice oxygen mechanisms,^54^ link catalyst magnetic properties to spin polarization responses, relax restrictive assumptions for chiral additives (e.g., 2D grids, binary dipoles), and replace heuristic averaging rules with bias-combination schemes grounded in a quantitative theory of CISS. Importantly, SA is not dictated solely by spin polarization: chirality and magnetic fields may also influence transport and kinetics through changes in conductivity, morphology, and microstructure, or via magnetohydrodynamic flows and bubble detachment.

Given the model’s simplicity, it is remarkable that it captures the range of trends observed experimentally. By introducing physical variables only where essential (e.g., dipole–dipole interactions in dense-additive regimes), the model yields several key insights: (1) a global chiral spin biase and magnetic field can be treated as effectively equivalent; (2) misaligned biases in heterochiral systems act as “weak links” that strongly reduce joint bias; (3) spatial heterogeneity allows global biases to enhance activity even when opposed; and (4) the dipole moment of chiral additives affects their enhancement of the OER activity. Although further refinements will increase predictive power, the conceptual simplicity of the current framework gives it significant illustrative value.

## Conclusions

This study demonstrates that the OER efficiency is affected by the nature, *B*_GSB_ versus *B*_LSB_, and magnitude of the spin polarization. Furthermore, it reveals intricacies of the interplay among different spin polarization types, illustrating how their interactions can either enhance or disrupt catalytic site selectivity through constructive or destructive interference. While OER serves as the primary case study, the findings can be extended to a broader range of radical-mediated reactions, including the oxygen reduction reaction, nitrogen fixation, and carbon dioxide reduction, all of which have been previously shown or proposed to be influenced by spin currents. By integrating insights from prior literature, experimental observations, and a statistical model, this work deepens our understanding of the fundamental mechanisms governing spin-controlled catalysis and provides a foundation for the rational design of more efficient spin-mediated catalysts.

## Supplementary Material



## Data Availability

Source code for the simulations is provided at github.com/wileyds/OER-modeling.

## Notes

Experimental details, synthesis and catalyst film preparation, characterization, electrochemical methods, model explaining the effect of global spin polarization induced by catalyst chirality or applied magnetic fields, equivalence of the effects of a magnetic field and global chiral bias, electrochemical measurements and spectroscopic characterization, model explanation for the effect of chiral additives near the catalyst surface, operational definition and spatial distribution of additives, assignment of orientation, enantiomer, and local spin bias to each additive, bias combination rule for sites influenced by multiple additives, dipole–dipole interactions between additives, resolution of distinct effects of additive dipole moment, combination of spin biases from chiral, and activation of magnetic catalysts (PDF)

## Symbol Definition Context

*A*, *A*_*IJ*_: dipole moment orientation of a chiral additive located in row *I* and column *J* in its lattice; symmetric binary variable with values +1 (upward) or −1 (downward), model variable
*B*_GSB_: global spin bias of arbitrary origin, model parameter
*B*_GSB,C_: global spin bias from catalyst chirality, model parameter
*B*_GSB,M_: global spin bias from an applied magnetic field, model parameter
*B*_*IJ*_: local spin bias produced by a chiral additive located in row *I* and column *J* in its lattice, model variable
|*B*_LSB_|: unsigned strength of the local spin bias from chiral additives, model parameter
*B*_GSB,net_: net global spin bias affecting all reaction intermediates equally, due to the joint effect of a magnetic field and inherent chirality of a catalyst, model variable
*B*_LSB,net_: net spin bias acting on a particular reaction intermediate due to the joint effect of several overlapping additives, model variable
*B*_net_: net spin bias acting on a particular reaction intermediate due to the joint effect of global and local spin biases, model variable
*B*_χ_: net spin bias acting on a particular reaction intermediate due to the joint effect of global and local spin biases from chiral sources only, model variable
CISS current: $\frac{J_{\mathrm{Enantiopure}}-J_{\mathrm{Achiral}}}{J_{\mathrm{Achiral}}}\times 100\%$ , where *J*_Enantiopure_ and *J*_Achiral_ correspond to the current density from chiral and achiral catalysts, experimental measurement
*d*: distance between adjacent sites in the lattice, model parameter
*D*: edge length of chiral additives’ domain of influence in number of lattice sites, model parameter
*E*, *E*_*IJ*_: enantiomeric type of the chiral additive located in row *I* and column *J* in its lattice: symmetric binary variable with values +1 (S) or −1 (R), model variable
*E*_↓_^0^: orientational energy associated with a chiral additive with dipole moment pointing downward, neglecting dipole–dipole interactions, model parameter
*E*_↑_^0^: orientational energy associated with a chiral additive with dipole moment pointing upward, neglecting dipole–dipole interactions, model parameter
ECSA: electrochemically accessible surface area, experimental measurement
*g*: circular dichroism dissymmetry factor, experimental measurement
*H*: applied magnetic field, experimental measurement
*H*_χ_: effective magnetic field associated with a spin bias from a chiral source, model variable
*i*: row index for sites in the reaction intermediate lattice, model index
*I*: row index for sites in the additive lattice, model index
*j*: column index for sites in the reaction intermediate lattice, model index
*J*: column index for sites in the additive lattice, model index
*J*_Achiral_: current density measured for an achiral catalyst, experimental measurement
*J*_Enantiopure_: current density measured for a chiral catalyst, experimental measurement
*J*_OFF_: catalyst current density measured without an applied magnetic field, experimental measurement
*J*_ON_: catalyst current density measured with an applied magnetic field, experimental measurement
*k*: dummy index, model index
*k*_B_: Boltzmann’s constant, physical constant
*m*: magnetic dipole moment of a reaction intermediate, model parameter
*M*: magnetization of an ensemble of reaction intermediates, model variable
*M*_∞_: saturation magnetization of an ensemble of reaction intermediates, model variable
Magnetocurrent: (*J*_ON_ – *J*_OFF_)/*J*_OFF_ × 100%, where *J*_ON_ and *J*_OFF_ correspond to the current response with and without an applied magnetic field at a given potential, experimental measurement
*n*: side length of a simulated patch of catalyst surface, model parameter
*N*: number of additives influencing the spin state of a specific reaction intermediate, model variable
*N*_↑_: number of upward-oriented reaction intermediates in a given lattice, model variable
*N*_↓_: number of downward-oriented reaction intermediates in a given lattice, model variable
*N*_T_: total number of reaction intermediates in a given lattice, model parameter
*o*_*ij*_: occupancy of a site in the reaction intermediate lattice at row *i* and column *j*: asymmetric binary random variable with value 0 (vacant) or 1 (occupied), model variable
*O*_*IJ*_: occupancy of a site in the additive lattice at row *I* and column *J*: asymmetric binary random variable with value 0 (vacant) or 1 (occupied), model variable
*P*_↑_: probability that a specific reaction intermediate is spin-up, model variable
*P*_↓_: probability that a specific reaction intermediate is spin-down, model variable
*P*_↑↓_: probability that spin-opposed reaction intermediates form a singlet product given the opportunity, model parameter
*P*_↑↑_: probability that spin-aligned reaction intermediates form a triplet product given the opportunity, model parameter
*r*: distance between two additives located at arbitrary points, model variable
*S*_A_: orientation of a chiral additive’s local spin bias, dictated by both its electric dipole moment orientation and enantiomeric type: symmetric binary random variable with values +1 (favors spin-up intermediates) or −1 (favors spin down intermediates), model variable
SA: specific activity, experimental measurement
*s*_*ij*_: spin state of a reaction intermediate located at row *i* and column *j* in its lattice: symmetric binary random variable with values +1 (spin up) or −1 (spin down), model variable
*T*: temperature, model parameter
*U*_DD_: interaction energy between two aligned electric dipoles, model variable
*U*_*IJ*_: net energy associated with a chiral additive’s orientation, incorporating both dipole–dipole interactions from other additives as well as contributions from other sources associated with the alignment factor α, model variable
*Y*_T_: triplet yield: number of triplet reaction products formed per 100 sites on a patch of catalyst surface, model variable
α: probability that a given chiral additive has its electric dipole moment oriented upward, model parameter
ε: probability that a given chiral additive is the S enantiomer (rather than R), model parameter
ϵ_0_: permittivity of free space, physical constant
ϵ_r_: dielectric constant/relative permittivity, model paramete
θ: occupation probability of sites in the reaction intermediate lattice, model parameter
Θ: occupation probability of sites in the chiral additive lattice, model parameter
μ: electric dipole moment of chiral additives, model parameter
ρ: weight factor used to calculate the net effect of combined global and local spin biases, model parameter
σ_*ij*_: joint spin-occupancy state of a reaction intermediate located at row *i* and column *j* in its lattice: ternary random variable with values +1 (occupied, spin-up), −1 (occupied, spin-down), or 0 (vacant), model variable

## References

[1] Masa J., Andronescu C., Schuhmann W. (2020). Electrocatalysis as the Nexus for
Sustainable Renewable Energy: The Gordian Knot of Activity, Stability,
and Selectivity. Angew. Chem., Int. Ed..

[2] Shi M.-M., Bao D., Yan J.-M., Zhong H.-X., Zhang X.-B. (2024). Coordination and
Architecture Regulation of Electrocatalysts for Sustainable Hydrogen
Energy Conversion. Acc. Mater. Res..

[3] Baglio V. (2021). Electrocatalysts
for Energy Conversion and Storage Devices. Catalysts.

[4] Liang Y., Banjac K., Martin K., Zigon N., Lee S., Vanthuyne N., Garcés-Pineda F. A., Galán-Mascarós J. R., Hu X., Avarvari N., Lingenfelder M. (2022). Enhancement of Electrocatalytic Oxygen
Evolution by Chiral Molecular Functionalization of Hybrid 2D Electrodes. Nat. Commun..

[5] Bloom B. P., Paltiel Y., Naaman R., Waldeck D. H. (2024). Chiral Induced Spin
Selectivity. Chem. Rev..

[6] Garcés-Pineda F. A., Yu J., Mesa C. A., Plana-Ruiz S., Ruano D., Liang Y., Lingenfelder M., Giménezb S., Galán-Mascarós J. R. (2025). Operando
evidence on the chirality-enhanced oxygen evolution reaction in intrinsically
chiral electrocatalysts. Chem. Sci..

[7] Wang Y., Sun J., Sun N., Zhang M., Liu X., Zhang A., Wang L. (2024). The spin polarization strategy regulates heterogeneous catalytic
activity performance: from fundamentals to applications. Chem. Commun..

[8] Chae K., Mohamad N. A. R. C., Kim J., Won D., Lin Z., Kim J., Kim D. H. (2024). The promise of chiral electrocatalysis for efficient
and sustainable energy conversion and storage: a comprehensive review
of the CISS effect and future directions. Chem.
Soc. Rev..

[9] Ren X., Wu T., Sun Y., Li Y., Xian G., Liu X., Shen C., Gracia J., Gao H.-J., Yang H., Xu Z. J. (2021). Spin-Polarized Oxygen
Evolution Reaction under Magnetic Field. Nat.
Commun..

[10] Wu T., Ren X., Sun Y., Sun S., Xian G., Scherer G. G., Fisher A. C., Mandler D., Ager J. W., Grimaud A., Wang J., Shen C., Yang H., Gracia J., Gao H.-J., Xu Z. J. (2021). Spin Pinning
Effect to Reconstructed
Oxyhydroxide Layer on Ferromagnetic Oxides for Enhanced Water Oxidation. Nat. Commun..

[11] Ren X., Wu T., Gong Z., Pan L., Meng J., Yang H., Dagbjartsdottir F. B., Fisher A., Gao H.-J., Xu Z. J. (2023). The Origin
of Magnetization-Caused Increment in Water Oxidation. Nat. Commun..

[12] Garcés-Pineda F. A., Blasco-Ahicart M., Nieto-Castro D., Lopez N., Galán-Mascarós J. R. (2019). Direct
Magnetic Enhancement of Electrocatalytic Water Oxidation in Alkaline
Media. Nat. Energy..

[13] Mtangi W., Tassinari F., Vankayala K., Jentzsch A. V., Adelizzi B., Palmans A. R. A., Fontanesi C., Meijer E. W., Naaman R. (2017). Control of
Electrons’ Spin Eliminates Hydrogen Peroxide Formation During
Water Splitting. J. Am. Chem. Soc..

[14] Barik D., Utkarsh U., Ghosh K. B. (2025). Spin-Controlled
Electrocatalysis:
An Out-of-the-Box Strategy for the Advancement of Electrochemical
Water Splitting. Chem. Commun..

[15] Ma S., Lee H., Moon J. (2024). Chirality-Induced
Spin Selectivity Enables New Breakthrough
in Electrochemical and Photoelectrochemical Reactions. Adv. Mater..

[16] Naaman R., Paltiel Y., Waldeck D. H. (2019). Chiral
Molecules and the Electron
Spin. Nat. Rev. Chem..

[17] Mtangi W., Kiran V., Fontanesi C., Naaman R. (2015). Role of the Electron
Spin Polarization in Water Splitting. J. Phys.
Chem. Lett..

[18] Vadakkayil A., Clever C., Kunzler K. N., Tan S., Bloom B. P., Waldeck D. H. (2023). Chiral Electrocatalysts Eclipse Water
Splitting Metrics
through Spin Control. Nat. Commun..

[19] Wang X., Yang Q., Singh S., Borrmann H., Hasse V., Yi C., Li Y., Schmidt M., Li X., Fecher G. H., Zhou D., Yan B., Felser C. (2025). Topological
Semimetals
with Intrinsic Chirality as Spin-Controlling Electrocatalysts for
the Oxygen Evolution Reaction. Nat. Energy.

[20] Sang Y., Tassinari F., Santra K., Zhang W., Fontanesi C., Bloom B. P., Waldeck D. H., Fransson J., Naaman R. (2022). Chirality
Enhances Oxygen Reduction. Proc. Natl. Acad.
Sci. U.S.A..

[21] Scarpetta-Pizo L., Venegas R., Barrías P., Muñoz-Becerra K., Vilches-Labbé N., Mura F., Méndez-Torres A. M., Ramírez-Tagle R., Toro-Labbé A., Hevia S., Zagal J. H., Oñate R., Aspée A., Ponce I. (2024). Electron Spin-Dependent
Electrocatalysis for the Oxygen Reduction Reaction in a Chiro-Self-Assembled
Iron Phthalocyanine Device. Angew. Chem., Int.
Ed..

[22] Zhang W., Ai J., Ouyang T., Yu L., Liu A., Han L., Duan Y., Tian C., Chu C., Ma Y., Che S., Fang Y. (2024). Chiral Nanostructured Ag Films for
Multicarbon Products
from CO_2_ Electroreduction. J. Am.
Chem. Soc..

[23] Pan H., Jiang X., Wang X., Wang Q., Wang M., Shen Y. (2020). Effective
Magnetic Field Regulation of the Radical Pair Spin States
in Electrocatalytic CO_2_ Reduction. J. Phys. Chem. Lett..

[24] Yang Y., Zhang L., Hu Z., Zheng Y., Tang C., Chen P., Wang R., Qiu K., Mao J., Ling T., Qiao S. Z. (2020). The Crucial Role
of Charge Accumulation
and Spin Polarization in Activating Carbon-Based Catalysts for Electrocatalytic
Nitrogen Reduction.. Angew. Chem. Int. Ed..

[25] Zhao Z., Wang D., Gao R., Wen G., Feng M., Song G., Zhu J., Luo D., Tan H., Ge X., Zhang W., Zhang Y., Zheng L., Li H., Chen Z. (2021). Magnetic-Field-Stimulated Efficient Photocatalytic N_2_ Fixation
over Defective BaTiO_3_ Perovskites. Angew. Chem., Int. Ed..

[26] Nazmutdinov R. R., Santos E., Schmickler W. (2013). Spin Effects
in Oxygen Electrocatalysis:
A Discussion. Electrochem. Commun..

[27] Hunt C., Zhang Z., Ocean K., Jansonius R. P., Abbas M., Dvorak D. J., Kurimoto A., Lees E. W., Ghosh S., Turkiewicz A., Garcés Pineda F. A., Fork D. K., Berlinguette C. P. (2022). Quantification of the Effect of an
External Magnetic Field on Water Oxidation with Cobalt Oxide Anodes. J. Am. Chem. Soc..

[28] Zou J., Zheng M., Li Z., Zeng X., Huang J. (2022). Magnetization
Triggered Oxygen Evolution Reaction Enhancement for Ferromagnetic
Materials. J. Mater. Sci.: Mater. Electron..

[29] Qin X., Teng J., Guo W., Wang L., Xiao S., Xu Q., Min Y., Fan J. (2023). Magnetic Field Enhancing OER Electrocatalysis
of NiFe Layered Double Hydroxide. Catal. Lett..

[30] Xu H., Qi J., Zhang Y., Liu H., Hu L., Feng M., Lü W. (2023). Magnetic Field-Enhanced
Oxygen Evolution Reaction via
the Tuneability of Spin Polarization in a Half-Metal Catalyst. ACS Appl. Mater. Interfaces.

[31] Bloom B. P., Graff B. M., Ghosh S., Beratan D. N., Waldeck D. H. (2017). Chirality
Control of Electron Transfer in Quantum Dot Assemblies. J. Am. Chem. Soc..

[32] Naaman R., Paltiel Y., Waldeck D. H. (2020). Chiral
Molecules and the Spin Selectivity
Effect. J. Phys. Chem. Lett..

[33] Amsallem D., Kumar A., Naaman R., Gidron O. (2023). Spin Polarization through
Axially Chiral Linkers: Length Dependence and Correlation with the
Dissymmetry Factor. Chirality.

[34] Varela S., Gutierrez R., Cuniberti G., Medina E., Mujica V. (2024). Chiral Spin
Selectivity and Chiroptical Activity in Helical Molecules. J. Chem. Phys..

[35] Jin Y., Fu W., Wen Z., Tan L., Chen Z., Wu H., Wang P.-P. (2024). Chirality Engineering
of Colloidal Copper Oxide Nanostructures
for Tailored Spin-Polarized Catalysis. J. Am.
Chem. Soc..

[36] Vadakkayil A., Dunlap-Shohl W. A., Joy M., Bloom B. P., Waldeck D. H. (2024). Improved
Catalyst Performance for the Oxygen Evolution Reaction under a Chiral
Bias. ACS Catal..

[37] Schroeder, D. V. An Introduction to Thermal Physics, 2000, Addison Wesley Longman, San Francisco.

[38] Clever C., Wierzbinski E., Bloom B. P., Lu Y., Grimm H. M., Rao S. R., Horne W. S., Waldeck D. H. (2022). Benchmarking Chiral
Induced Spin Selectivity Measurements Towards Meaningful Comparisons
of Chiral Biomolecule Spin Polarizations. Isr.
J. Chem..

[39] Wei J., Bloom B. P., Dunlap-Shohl W. A., Clever C. B., Rivas J. E., Waldeck D. H. (2023). Examining the Effects
of Homochirality for Electron
Transfer in Protein Assemblies. J. Phys. Chem.
B.

[40] Dean, J. A. Lange’s Handbook of Chemistry, 15th ed.; McGraw-Hill: New York, 1999.

[41] Guan X., Wang M., Chen Z., Cao C., Li Z., Xue R., Fu Y., Johannessen B., Tadich A., Yi J., Fan H., Wang N., Jia B., Li X., Ma T. (2025). Creating Spin
Channels in SrCoO_3_ through Trigonal-to-Cubic Structural
Transformation for Enhanced Oxygen Evolution/Reduction Reactions. Angew. Chem., Int. Ed..

[42] Tkalych A. J., Yu K., Carter E. A. (2015). Structural and Electronic Features of β-Ni­(OH)_2_ and β-NiOOH from First Principles. J. Phys. Chem. C.

[43] Harada M., Saito A., Nakahira H., Mori Y., Kawaguchi S. (2025). In Situ Observations
of Catalytically Active Sites of Cobalt–Manganese Spinel Oxides
as Efficient Bifunctional Electrocatalysts for Oxygen Evolution and
Reduction Reactions. ACS Appl. Energy Mater..

[44] Cheng H., Yan D., Wu L., Liang P., Cai Y., Li L. (2022). Chemical Characterization,
Absolute Configuration, and Optical Purity of (1S)-(+)- and (1R)-(−)-10-Camphorsulfonic
Acid. Acta Crystallogr. Sect. C:Struct. Chem..

[45] Brunelli M., Fitch A. N., Mora A. J. (2002). Low-Temperature
Crystal Structure
of S-Camphor Solved from Powder Synchrotron X-ray Diffraction Data
by Simulated Annealing. J. Solid State Chem..

[46] Lu Z., Manias E., Macdonald D. D., Lanagan M. (2009). Dielectric Relaxation
in Dimethyl Sulfoxide/Water Mixtures Studied by Microwave Dielectric
Relaxation Spectroscopy. J. Phys. Chem. A.

[47] Basma N. S., Headen T. F., Shaffer M. S. P., Skipper N. T., Howard C. A. (2018). Local Structure
and Polar Order in Liquid N-Methyl-2-pyrrolidone (NMP). J. Phys. Chem. B.

[48] Ghosh S., Bloom B. P., Lu Y., Lamont D., Waldeck D. H. (2020). Increasing
the Efficiency of Water Splitting through Spin Polarization Using
Cobalt Oxide Thin Film Catalysts. J. Phys. Chem.
C.

[49] Zhu J., Peng X., Xi P., Jia C., Gao D. (2025). Dual-Channel
Regulation of Spin Polarization Achieves 1 + 1 > 2 Electrocatalytic
Performance in Spinel Ferrites. Nano Lett..

[50] Nair A. N., Fernandez S., Marcos-Hernández M., Romo D. R., Singamaneni S. R., Villagran D., Sreenivasan S. T. (2023). Spin-Selective
Oxygen Evolution Reaction in Chiral Iron Oxide Nanoparticles: Synergistic
Impact of Inherent Magnetic Moment and Chirality. Nano Lett..

[51] Naaman R., Waldeck D. H. (2012). Chiral-Induced Spin Selectivity Effect. J. Phys. Chem. Lett..

[52] Göhler B., Hamelbeck V., Markus T. Z., Kettner M., Hanne G. F., Vager Z., Naaman R., Zacharias H. (2011). Spin Selectivity
in Electron Transmission Through Self-Assembled Monolayers of Double-Stranded
DNA. Science.

[53] Kettner M., Maslyuk V. V., Nürenberg D., Seibel J., Gutierrez R., Cuniberti G., Ernst K.-H., Zacharias H. (2018). Chirality-Dependent
Electron Spin Filtering by Molecular Monolayers of Helicenes. J. Phys. Chem. Lett..

[54] He Y., Kang Z., Li J., Li Y., Tian X. (2023). Recent progress
of manganese dioxide-based electrocatalysts for the oxygen evolution
reaction. Ind. Chem. Mater..

