# Supplementary material for: Synergistic Spin-Mediated Catalysis for the Oxygen Evolution Reaction
Source: J Am Chem Soc. 2025 Nov 10;147(46):42659–69. doi: 10.1021/jacs.5c14127 (PMC12636008; doi:10.1021/jacs.5c14127)
Supplement: Supplementary file 1 [file ja5c14127_si_001.pdf]

Supplemental information for:

Synergistic Spin-Mediated Catalysis for the Oxygen Evolution Reaction

Aravind Vadakkayil, Fiham Fahim, Wiley A. Dunlap-Shohl, Michael Vullo, Brian P. Bloom,* David H. Waldeck*

Chemistry Department, University of Pittsburgh, Pittsburgh, Pa 15260, USA.

Email: bpb8@pitt.edu, dave@pitt.edu

Table of Contents

Methods

Materials

Synthesis and Catalyst Film Preparation

Characterization

Electrochemical Methods

Dipole Moment Calculation of Camphorsulfonic Acid (CSA)

Supplemental Figures and Discussion

Supplementary Note 1: Model Explaining the Effect of Global Spin Polarization Induced by Catalyst Chirality or Applied Magnetic Fields.

Supplementary Note 2: Equivalence of the Effects of a Magnetic Field and Global Chiral Bias.

Supplementary Note 3: Electrochemical Measurements and Spectroscopic Characterization

Supplementary Note 4: Model Explanation for the Effect of Chiral Additives Near the Catalyst Surface.

4.1 Operational Definition and Spatial Distribution of Additives.

4.2 Assignment of Orientation, Enantiomer, and Local Spin Bias to Each Additive.

4.3 Bias Combination Rule for Sites Influenced by Multiple Additives.

4.4 Dipole-Dipole Interactions Between Additives.

4.5 Resolution of Distinct Effects of Additive Dipole Moment.

Supplementary Note 5: Combination of Spin Biases from Chiral and Magnetic Sources.

Supplementary Note 6: Activation of Magnetic Catalysts

List of Variables

References

Methods

Materials – All commercial materials and solvents were used without purification unless otherwise indicated. Sodium borohydride, L-, D-, DL-cysteine, sodium citrate, cobalt (II) chloride, iron (III) chloride hexahydrate, S-camphor sulfonic acid, R-camphor sulfonic acid, S-Limonene, R-Limonene, S-camphor, R-camphor, and Nafion perfluorinated resin solution were purchased from Sigma-Aldrich. NaOH was purchased from Fisher Scientific.

Synthesis and Catalyst Film Preparation

Synthesis of Co_3O_4 Catalysts – Undoped Co_3O_4 nanoparticles were synthesized using a previously established method.[1,2] In a typical procedure, 15 mL of deionized water was combined with 2.5 mL of 100 mM $NaBH_4$, 2 mL of 100 mM L-, D-, or DL-cysteine, 2 mL of 100 mM sodium citrate, and 1 mL of 200 mM cobalt (II) chloride in a round-bottom flask. The mixture was continuously stirred at room temperature (23–25°C) for 2 hours. During this process, the solution gradually turned into a transparent dark brown color, indicating the successful formation of Co_3O_4 nanoparticles. The resulting nanoparticles were purified by adding isopropanol in a sevenfold excess, leading to precipitation, followed by centrifugation at 8000 rpm for 20 minutes. The collected nanoparticles were dried and subsequently re-dispersed in water for further use.

Synthesis of $Fe_{0.7}Co_{2.3}O_4$ Catalysts – Fe-doped Co_3O_4 nanoparticles were synthesized by incorporating 1 mL of 46 mM iron (III) chloride hexahydrate into the precursor solution. The synthesis process and purification of doped nanomaterial was the same as that of undoped cobalt oxide.

Preparation of Catalyst Films – Undoped, and doped cobalt oxide ink solutions were prepared by mixing 0.5 mg of nanomaterial with 12.5 μL of 5 wt% Nafion perfluorinated resin solution and 250 μL of water/isopropyl alcohol (3:1 v/v). The mixture was sonicated for 15 minutes and a homogeneous dispersion formed. A 1 μL aliquot was drop cast onto a mechanically polished 0.07 cm^2 glassy carbon electrode and then dried in an oven at 70°C for 30 min to evaporate the solvents.

Preparation of Catalyst Films with additives – Undoped or doped cobalt oxide ink solutions were prepared by mixing a specific concentration of S-additive (CSA/camphor/limonene) or a racemic mixture of the additive (CSA/camphor/limonene) into 12.5 μL of 5 wt% Nafion perfluorinated resin solution and 250 μL of water/isopropyl alcohol (3:1 v/v). The mixture was sonicated for 15 minutes and then 0.5 mg of the catalyst was added to the solution and sonicated for an additional 30 minutes. Once a homogeneous dispersion was formed a 1 μL aliquot was drop cast onto a mechanically polished 0.07 cm^2 glassy carbon electrode and then dried in an oven at 70°C for 30 min to evaporate the solvents.

Characterization

Circular dichroism measurements were performed using a Jasco J810 CD spectropolarimeter with an integration time of 4 s and a bandwidth of 1 nm. UV-Vis absorbance measurements were made using an Agilent (model Cary 60 UV-Vis) spectrometer.

Electrochemical Methods

Electrochemical Measurements – Electrochemical experiments were carried out using a 618B or 420A (CH Instruments) potentiostat in a 1 M NaOH electrolyte solution, using an Ag|AgCl reference electrode (CHI) and Pt mesh as the counter electrode, unless otherwise specified. Linear sweep voltammetry experiments were collected at a scan rate of 10 mV/s. The electrochemical results reported in this work were *iR* compensated using the CH instruments software package command prior to measurements.

For the determination of electrochemical surface area, ECSA, cyclic voltammograms were taken in a non-Faradaic region (0.45 to 0.5 V vs. Ag|AgCl reference electrode) at scan rates from 10 to 70 mV/s. The ECSA was calculated using the formula; ECSA = C_{dl}/C_s where C_{dl} is the double layer capacitance obtained from the slope of the current (charging current at a constant potential) versus scan rate plot, and C_s is the specific capacitance of the material; 40 µF/cm^2.[3] Specific activity was determined using the following equation: Specific activity = J_{geo}/RF, where J_{geo}, and RF refer to the geometric current density at 350 mV overpotential, and roughness factor, respectively and RF = ECSA/geometric area of the electrode.

Potentiostatic bulk electrolysis – Prior to investigating the effects of an external magnetic field, the cobalt oxide catalyst films, drop-cast onto glassy carbon substrates, were pre-treated by applying a constant potential of 2.0 V vs RHE for 15 minutes in 1 M NaOH. This electrochemical conditioning step served to stabilize the catalyst surface and promote magnetic activation. Following this treatment, the influence of an applied magnetic field on the specific activity of the catalyst was systematically evaluated.

Dipole Moment Calculation of Camphorsulfonic Acid (CSA)

The dipole moment of camphorsulfonic acid (CSA) was determined using Avogadro 1.2.0.

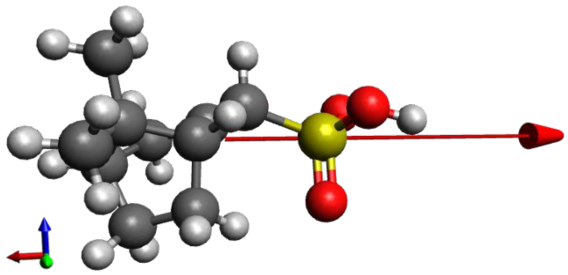

Scheme S1: Molecular structure of camphor sulfonic acid (CSA) drawn using Avogadro 1.2.0. The red arrow represents the direction of the calculated dipole moment.

The CSA molecule was constructed in the software, its geometry was optimized, and the molecular dipole moment was obtained from the molecular properties panel.

Supplemental Figures and Discussion

Supplementary Note 1: Model Explaining the Effect of Global Spin Polarization Induced by Catalyst Chirality or Applied Magnetic Fields.

We begin constructing our model using the simplest case, in which a uniform tendency to polarize reaction intermediate spins is applied across the surface of the catalyst, arising from either an external magnetic field or the intrinsic chirality of the catalyst itself (i.e., a global spin

bias/GSB acting alone). Let a patch of catalyst surface be represented by an $n \times n$ square lattice representing adsorption sites for reaction intermediates (in most simulations, we will take n = 20 except when visualizing sample lattices, where we will use n = 12 for ease of presentation). Further assume that these sites are randomly occupied by reaction intermediates that have an average fractional coverage θ. The occupancy o_{ij} of the site in the ith row and j^{th} column of the lattice is thus a random variable that takes the value 0 if the site is vacant and 1 if the site is filled, according to a binomial distribution with probability parameter θ. In this manner, we generate random lattices describing the positions of adsorbed reaction intermediates. Example lattices are shown schematically in Figure S1.

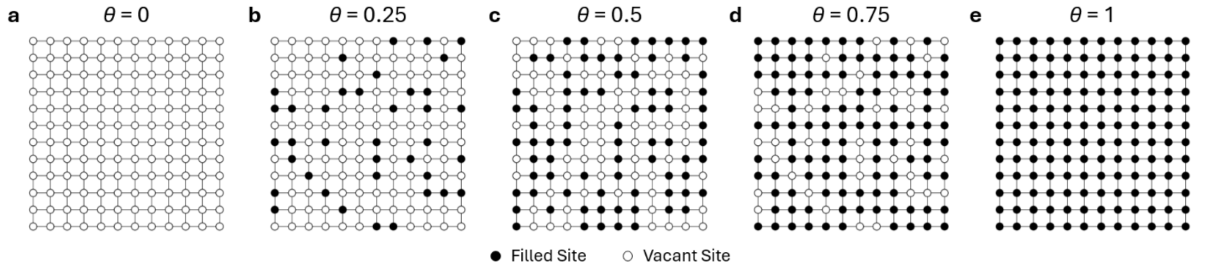

Figure S1. Occupancy of reaction intermediates in a square lattice representing a patch of catalyst surface, wherein any given site has a 0% (a), 25% (b), 50% (c), 75% (d), or 100% (e) probability of being filled. Occupied sites ($o_{ij} = 1$) are indicated by black circles and unoccupied ones ($o_{ij} = 0$) by white circles.

The spin state s_{ij} at a given site may be up (+1) or down (-1), and random spin lattices may be generated in an analogous manner to the occupancy, using a binomial distribution with a different parameter, B_{GSB}, which describes the probability that a given site will be spin-up (strictly, this probability is 0.5 + B_{GSB}, such that positive values of B_{GSB} correspond to spin-up preference and negative values to spin-down). We hereafter refer to B_{GSB} as a *global spin bias*, and it arises from the catalyst's chirality, an applied magnetic field, or the net result of both. The spin and occupancy states can be multiplied at each site to give a third variable $\sigma_{ij} = s_{ij}$ o_{ij} that takes on a value of 0 if site (i, j) is vacant, +1 if the site is occupied by a spin-up reaction intermediate, or -1 if it is occupied by a spin-down reaction intermediate. Examples of representative spin lattices for various values of B_{GSB} are shown in Figure S2.

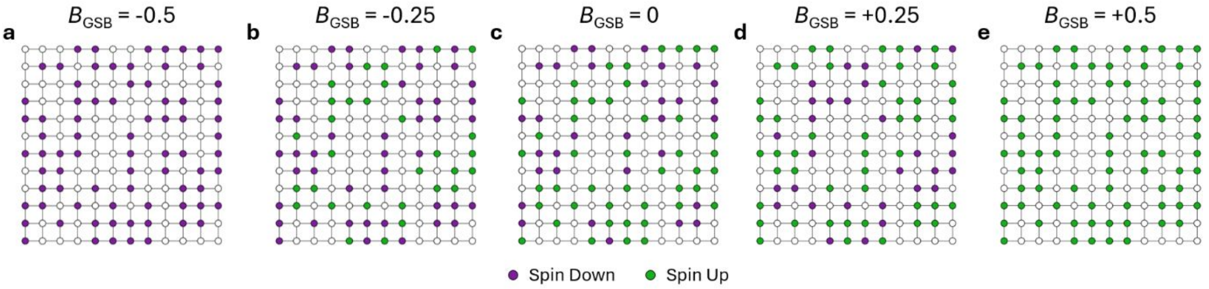

Figure S2. Spin states of the lattice generated in Figure S1c (50% reaction intermediate occupancy), with spin states assigned to the filled sites using uniform downward (a), moderate downward (b), neutral (c), moderate upward (d), and uniform upward (e) global spin biases. Green circles indicate spin-up reaction intermediates ($\sigma_{ij} = +1$), purple circles indicate spin-down reaction intermediates ($\sigma_{ij} = -1$) , and white circles indicate vacant sites ($\sigma_{ij} = 0$).

Having outlined a way to describe the behavior of a random lattice of spin-polarizable reaction intermediates under the influence of a chiral bias, we now proceed to describe the

possible outcomes of reactions between those intermediates. Two possible types of reaction products are considered: *triplets*, which can form from two adjacent intermediates that have their spins aligned, and *singlets*, which can form if the intermediates are opposed. We identify the triplets as the desired product (i.e., O_2), and singlets as unwanted byproducts such as H_2O_2. The principal figure of merit that our model produces is therefore calculated as the triplet yield Y_T, expressed as the number of triplet reaction products formed per 100 sites on a patch of catalyst surface. For each filled site, a nearest neighbor is chosen at random (if present), and is given an opportunity to "react." (Note that we employ a periodic boundary condition such that sites on an edge of the lattice are treated as nearest neighbors of those on the opposite edge.) If the neighbor is spin-aligned, the outcome of this reaction is determined by drawing from a binomial distribution with probability $P_{\uparrow\uparrow}$; otherwise, a random draw is carried out with probability $P_{\uparrow\downarrow}$. This process is performed until either a "reaction" occurs or all available neighbors have been tried. If a reaction occurs, the intermediate and its paired neighbor are removed from further consideration. If no reaction occurs, the intermediate and its neighbors remain unpaired (but may have further opportunities to react later when their neighbors are chosen). After this pairing algorithm concludes, the triplet and singlet yields are determined by counting the respective pairs of reaction products. The results of this pairing algorithm for a sample lattice with no spin bias (B_{GSB} = 0) are shown in Figure S3 for different values of $P_{\uparrow\uparrow}$ and $P_{\uparrow\downarrow}$.

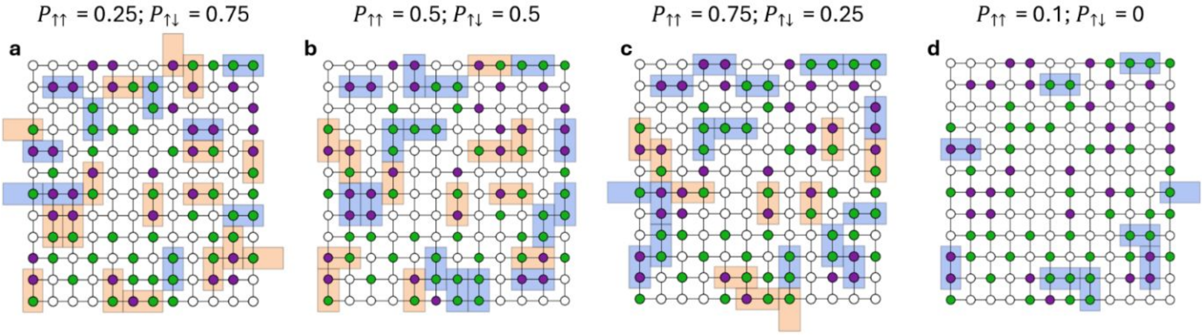

Figure S3. Results of the pairing algorithm that assigns singlet and triplet reaction products to possible intermediate pairs in the randomly generated lattice shown in Figure S2c (B_{GSB} = 0), with singlets intrinsically more favorable than (a), equally favorable as (b), less favorable than (c) triplet products, or completely forbidden (d). The latter condition represents the parameter combination that best corresponds to our experiments, which are performed at high pH and therefore unfavorable conditions for singlet formation. Spin-up and spin-down reaction intermediates are depicted as green and purple circles and vacant sites by white circles, as above; singlet and triplet products are indicated by orange and blue boxes. Note that not all sites that can form triplet or singlet products do form them; only the latter are indicated by the blue and orange boxes and are counted in the determination of triplet and singlet yields. Where the boxes hang off the edge of the grid, the enclosed reaction intermediates should be understood to pair with the corresponding site on the opposite edge via the periodic boundary condition.

In the manner developed above, we can calculate triplet yields from randomly generated patches of catalyst using only four probability parameters – surface coverage θ, global spin bias B_{GSB}, and triplet and singlet formation probabilities $P_{\uparrow\uparrow}$ and $P_{\uparrow\downarrow}$. We interrogate the effects of a chiral bias on the OER activity by varying these parameters and monitoring their effects on the triplet and singlet yields. To better approximate the behavior of a real catalyst, we generate an ensemble of 1000 such lattices for each combination of parameters of interest,

allowing us to calculate statistics on the resulting distributions of triplet and singlet yields. The results of sample calculations are shown in Figure S4, where we have chosen to fix $\theta = 0.5$ to represent a condition in which most of the adsorption sites on the catalyst surface are filled but which still has some vacancies, allow B_{GSB} to vary from -0.5 to +0.5 in increments of 0.1, and let $P_{\uparrow\uparrow}$ and $P_{\uparrow\downarrow}$ independently take on values in the set {0.25, 0.5, 0.75} to represent a range of cases in which both triplets and singlets could be heavily favored or disfavored. We find in all cases that the mean triplet yield increases nearly parabolically ($R^2 > 0.998$) as the chiral bias diverges from the neutral value of 0 that corresponds to no net preference for either spin-up or spin-down reaction intermediates. Two features of this relationship bear special emphasis. First, the symmetric nature indicates that the sign of the spin is not important; catalytic activity is improved as long as the spins of intermediates are similar to those of their nearest neighbors. Second, the curvature of the graph demonstrates that the benefits of chirality (or applied magnetic field) do not set in immediately once the catalyst symmetry is broken – a weakly chiral catalyst is barely distinguishable from a completely achiral one. The more chiral the catalyst becomes, the greater the marginal gains in OER activity. A corollary observation is that small deviations from a highly ordered spin lattice have disproportionately large effects on catalytic activity. If we allow $P_{\uparrow\uparrow}$ and $P_{\uparrow\downarrow}$ to vary, this overall behavior is maintained, but the minima and maxima of the triplet yield curves shift in response to the product favorabilities.

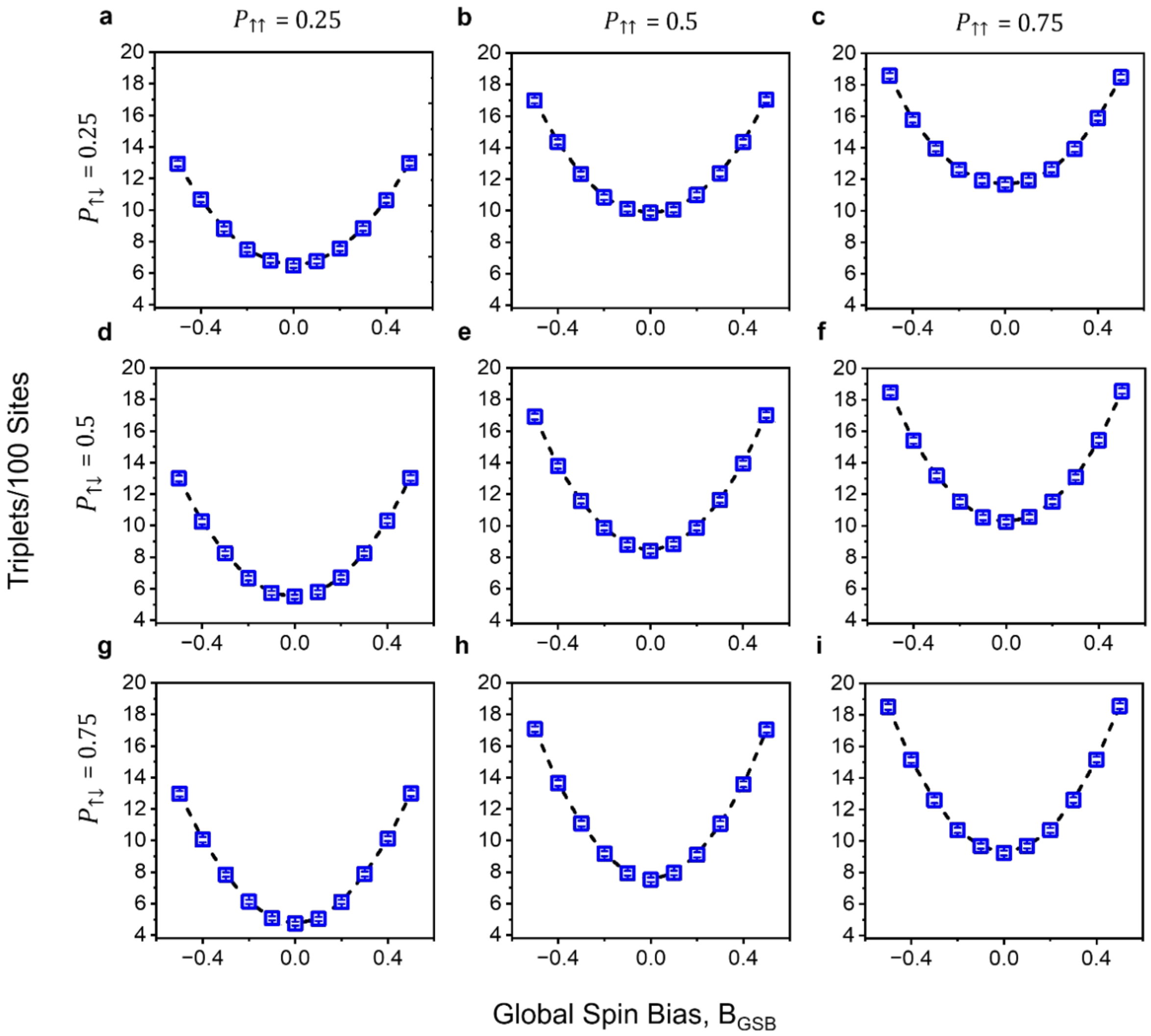

Figure S4. Results of Monte Carlo simulations of triplet yield and triplet selectivity of an OER catalyst

in response to changing chiral bias or applied magnetic field, with reaction intermediate coverage parameter θ = 0.5 and various combinations of triplet and singlet reaction rate parameters $P_{\uparrow\uparrow}$ and $P_{\uparrow\downarrow}$. Each data point represents the mean of the outcomes from 1000 independent simulations corresponding to the same combination of parameters, and the error bars represent 95% confidence intervals about this mean; however, these may appear visually compressed due to the larger size of the data markers. Dashed curves correspond to parabolic fits of the simulated data, for which $R^2 > 0.998$ in all cases.

Although we characterize model uncertainty using the 95% confidence interval to the mean, it is useful to understand the spread in the raw output of the model. In Figure S5, we display histograms of triplet yield for three selected simulations of catalysts under different global biases, holding the other parameters fixed at $\theta = 0.5$, $P_{\uparrow\downarrow} = 0$, and $P_{\uparrow\uparrow} = 0.1$ (i.e., corresponding to the simulations displayed in Figure 3). Visual inspection of the histograms indicates that the triplet yield follows an approximately normal distribution, with a FWHM of ~2-3 triplets/100 sites.

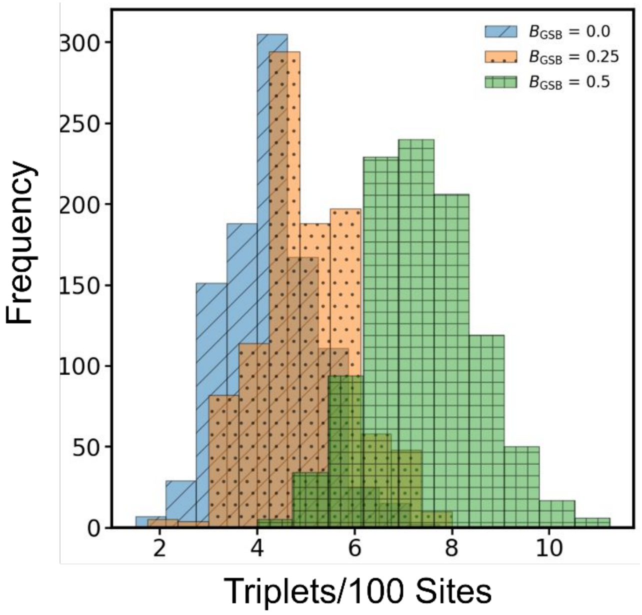

Figure S5. Histograms of triplet yield for simulations of catalysts performed at different global biases, with $\theta = 0.5$ and $P_{\uparrow\downarrow} = 0$, and $P_{\uparrow\uparrow} = 0.1$, respectively.

Supplementary Note 2: Equivalence of the Effects of a Magnetic Field and Global Chiral Bias.

The lattice of individual reaction intermediates is conceptually equivalent to an ensemble of spins belonging to a two-state paramagnet. Each spin has one of two possible states: either upward – i.e., oriented with the magnetic field (or chiral bias), or downward – i.e., against it. In the former state, the energy is $-mH$, where m is the magnetic dipole moment of the intermediate and H is the applied or effective magnetic field. Following Boltzmann statistics, the probabilities of the up and down states are therefore[4]

$$P_\uparrow = \frac{\exp\left(\frac{mH}{k_B T}\right)}{\exp\left(\frac{mH}{k_B T}\right) + \exp\left(-\frac{mH}{k_B T}\right)} \qquad P_\downarrow = \frac{\exp\left(-\frac{mH}{k_B T}\right)}{\exp\left(\frac{mH}{k_B T}\right) + \exp\left(-\frac{mH}{k_B T}\right)},$$

where k_B is Boltzmann's constant and T is temperature. The fractional magnetization of the ensemble (i.e., relative to its saturation value M_∞) is equal to the expectation value of upward spins minus that of the downward spins, divided by the total number of spins N_T:

$$\frac{M}{M_\infty} = \frac{\langle N_\uparrow \rangle - \langle N_\downarrow \rangle}{\langle N_\uparrow \rangle + \langle N_\downarrow \rangle} = \frac{N_T P_\uparrow - N_T P_\downarrow}{N_T} = P_\uparrow - P_\downarrow$$

$$\frac{M}{M_\infty} = \frac{\exp\left(\frac{mH}{k_B T}\right) - \exp\left(-\frac{mH}{k_B T}\right)}{\exp\left(\frac{mH}{k_B T}\right) + \exp\left(-\frac{mH}{k_B T}\right)} = \tanh\left(\frac{mH}{k_B T}\right)$$

Since $P_\downarrow = 1 - P_\uparrow$,

$$P_\uparrow - P_\downarrow = 2P_\uparrow - 1 = \tanh\left(\frac{mH}{k_B T}\right)$$

Recalling the definition of a spin bias as an enhancement to the spin-up probability – i.e., $P_\uparrow = \frac{1}{2} + B_{GSB}$, we have

$$B_{GSB} = \frac{1}{2}\tanh\left(\frac{mH}{k_B T}\right)$$

Hence, the bias is equal to half the normalized magnetization of the spin intermediate ensemble. Since M/M_∞ ranges between -1 and 1, B_{GSB} takes on values between -½ and ½, which correctly allows $P_\uparrow$ to range from 0 to 1. Note that since the hyperbolic tangent is a monotonically increasing function for all real values of H, this framing allows us to calculate a unique effective magnetic field associated with any spin bias by inverting the above expression.

Supplementary Note 3: Electrochemical Measurements and Spectroscopic Characterization

Figure S6 shows representative LSVs of S- (orange), R- (green) and rac-additives (blue) in equal concentrations, as well as the case without additives (black) to $Fe_{0.7}Co_{2.3}O_4$ catalyst in solutions in Nafion. The experiments are consistent with that of our previous report[5] and imply that the enantiopurity of chiral additives is the important criterion for improved OER activity and not the particular enantiomorph.

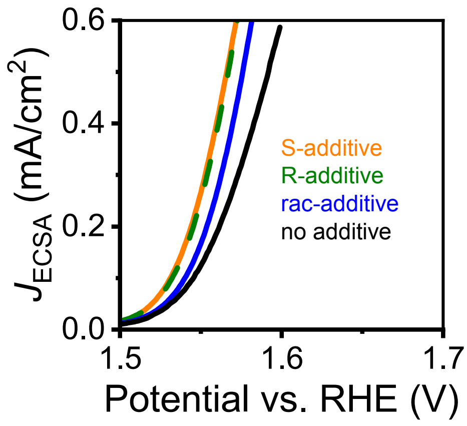

Figure S6. Linear sweep voltammograms of $Fe_{0.7}Co_{2.3}O_4$ catalyst ink suspensions in Nafion with S-CSA (orange), R-CSA (green), racemic-CSA (bue), and no-CSA (black) in 1M NaOH.

Figure S7 shows representative double layer capacitance measurements from 10 to 70 mV s^{-1} of $Fe_{0.7}Co_{2.3}O_4$ catalysts with (**a**) CSA, (**b**) camphor, and (**c**) limonene, with S-CSA additives (green), rac-CSA additives (orange), and without additives (black) for determining the electrochemical surface area. The filled symbols represent the double layer capacitance when the potential is swept anodically. Table S1 summarizes the experimentally determined ECSA of the catalyst films with different additives. The similarity in ECSA values with and without additives indicate that the additives do not adsorb onto the active sites of the catalyst.

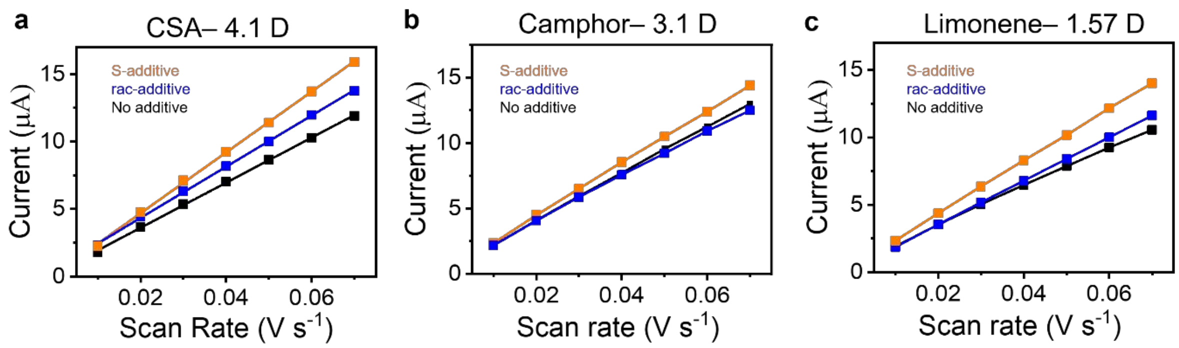

Figure S7. Representative double layer capacitance measurements of $Fe_{0.7}Co_{2.3}O_4$ catalysts with (**a**) CSA, (**b**) camphor, and (**c**) limonene with S-additives (orange), rac-additives (blue), and without additives (black). The solid line is a linear fit to the data.

Table S1. ECSA values for different catalyst films in different binder-additive combinations. The data represents ECSA averages and error bars of three independently prepared electrodes.

	**S-additive**	**rac-additive**	**No additive**
**Camphor sulfonic acid**	5.4 ± 0.12 cm^2	5.0 ± 1.0 cm^2	3.5 ± 0.5 cm^2
**Camphor**	3.4 ± 0.7 cm^2	3.0 ± 1.0 cm^2	3.0 ± 1.0 cm^2
**Limonene**	4.80 ± 0.10 cm^2	4.52 ± 0.11 cm^2	4.10 ± 0.2 cm^2

In order to verify that the improved activity in OER with chiral additives is not associated with imprinting chirality onto the catalysts, spectroscopic measurements on catalyst ink solutions were performed; see Figure S8. Figs. S8**a** and S8**b** show absorbance and circular dichroism, respectively, of solutions comprising $Fe_{0.7}Co_{2.3}O_4$ (black), S-CSA (red), S-camphor (green), S-limonene (orange) and $Fe_{0.7}Co_{2.3}O_4$ with S-CSA (blue), $Fe_{0.7}Co_{2.3}O_4$ with S-camphor (purple), and $Fe_{0.7}Co_{2.3}O_4$ with S-limonene (brown), in Nafion. No Cotton effects are observed in the catalyst absorption regions for solutions prepared with chiral additives. However, a sharp transition appears at approximately 325 nm consistent with the results from control experiments containing only the chiral additives (Figure S8b). These data imply that the CSA/camphor/limonene is not directly imprinting chirality onto the electronic states of the catalyst; i.e., no new features emerge in regions associated with electronic transitions of the catalysts. Please note that in our previous work,[1] with L-cysteine passivated $Fe_{0.7}Co_{2.3}O_4$ catalysts, the cysteine imprints chirality onto the electronic states of the catalyst, resulting in Cotton effects at the Co(II) → Co(III) intra-particle and surface state (including ligand) → Co(III) optical transitions of the $Fe_{0.7}Co_{2.3}O_4$. The distinct differences between achiral-$Fe_{0.7}Co_{2.3}O_4$ with S-CSA/camphor/limonene and L-cysteine passivated $Fe_{0.7}Co_{2.3}O_4$ from our previous work clearly demonstrates that the addition of chiral additives does not imprint chirality onto the catalyst's electronic properties. Figure S8**c** and S8**d** show absorbance and circular dichroism of solutions comprising L- (purple), D- (violet), rac-(black) Co_3O_4 catalyst ink suspension in Nafion.

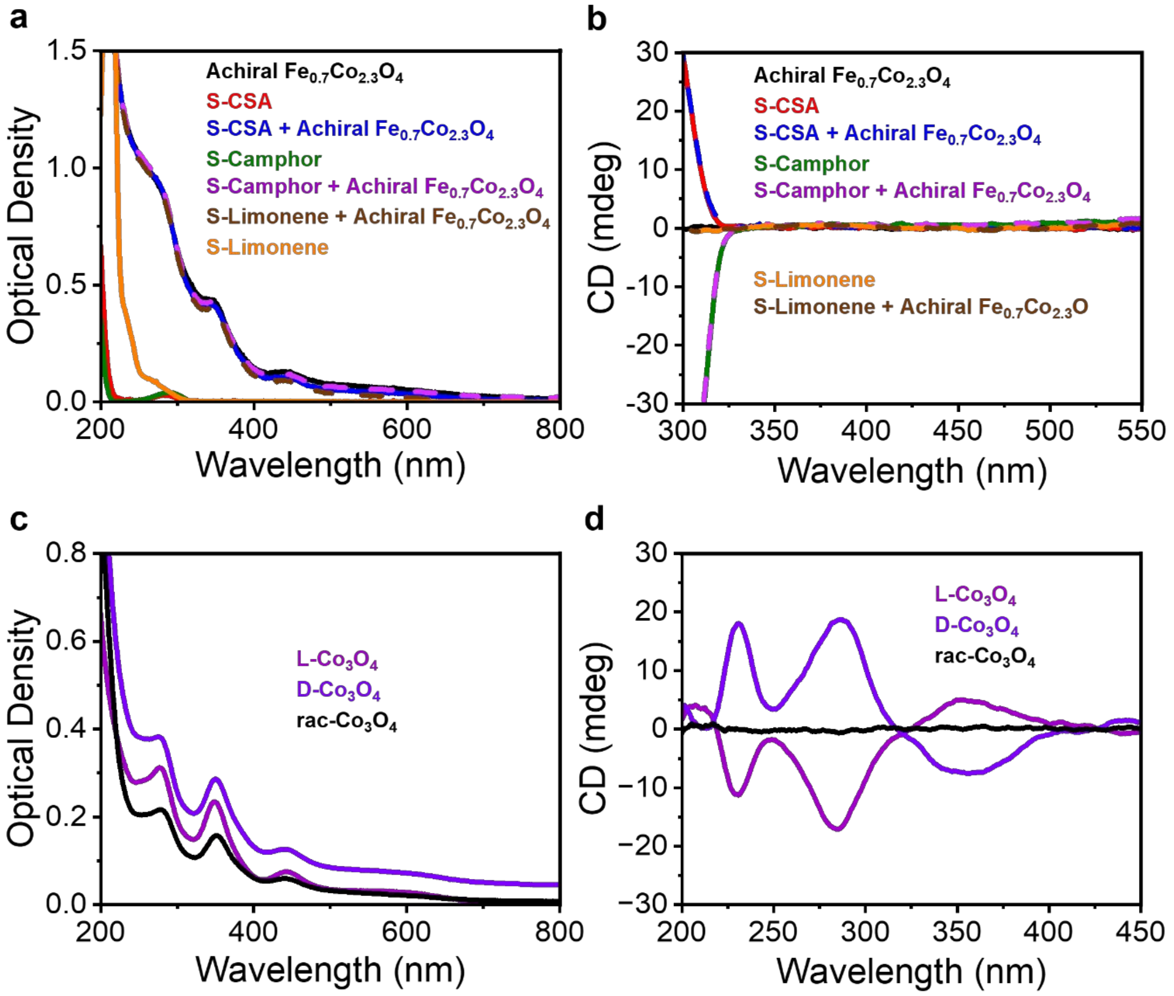

Figure S8. Spectroscopic characterization of chiral imprinting. Panels **a** and **b** show absorbance and circular dichroism of solutions comprising $Fe_{0.7}Co_{2.3}O_4$ (black), S-CSA (red), S-camphor (green), S-limonene (orange) and $Fe_{0.7}Co_{2.3}O_4$ with S-CSA (blue), $Fe_{0.7}Co_{2.3}O_4$ with S-camphor (purple), $Fe_{0.7}Co_{2.3}O_4$ with S-limonene (brown), in Nafion. Panels **c** and **d** show data for absorbance and circular dichroism of solutions comprising L- (purple), D- (violet), rac-(black) Co_3O_4 catalyst ink suspension in Nafion.

To highlight that additives (CSA, camphor, and limonene) do not contribute to the observed current under OER potentials, a series of control experiments were conducted. Figure S9 **a, b**, & **c** shows cyclic voltammograms of bare glassy carbon electrodes (GCE) in basic (1M NaOH) conditions with (blue) and without (green) adding 20 mM of CSA, camphor, and limonene, respectively in the electrolyte solution. The currents are approximately the same for both systems. To ensure that the catalyst does not facilitate additive oxidation, additional experiments were also performed on $Fe_{0.7}Co_{2.3}O_4$ (Panel **d, e**, & **f**) coated electrodes with (blue) and without (green) 20 mM of additive (CSA/camphor/limonene) concentration in the electrolyte solution. No changes to the current response are observed upon introduction of the additives and imply additives do not contribute to the current response during OER.

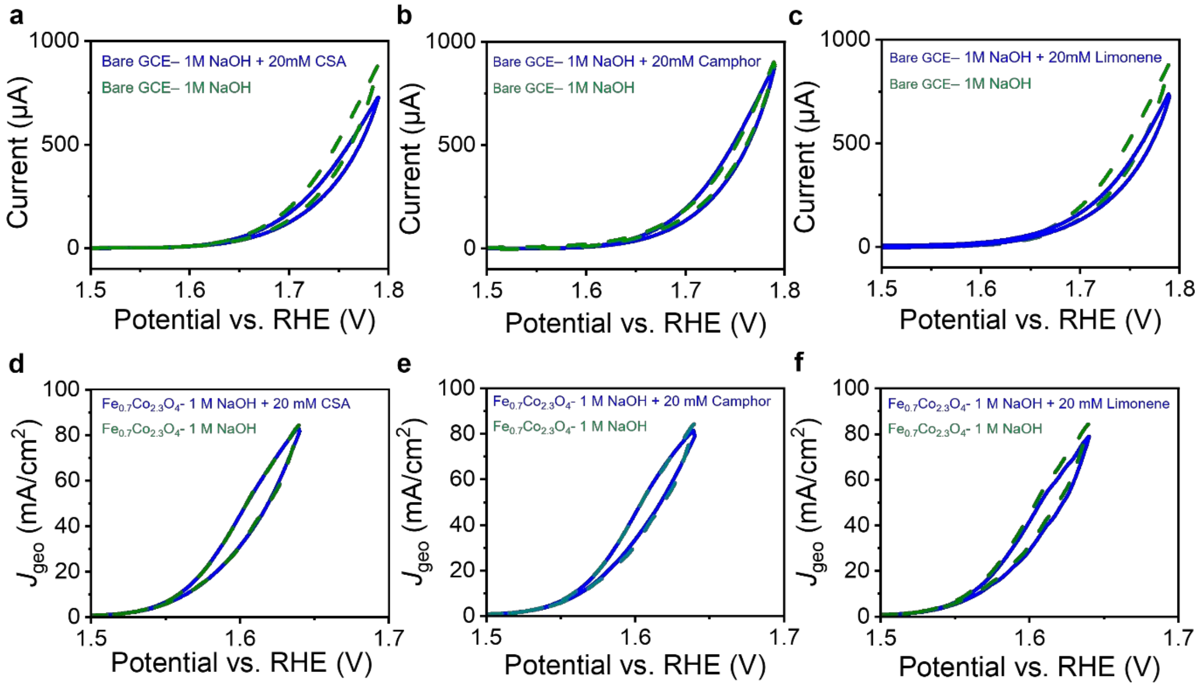

Figure S9. Cyclic voltammograms of a bare glassy carbon electrode in 20 mM CSA (**a**), 20 mM camphor (**b**), and 20 mM limonene (**c**) solution with (blue solid line) and without (dashed green line) the additive. Panels (**d, e and f**) plot representative voltammograms of $Fe_{0.7}Co_{2.3}O_4$ catalysts with (blue solid line) and without (dashed-green line) 20 mM CSA, camphor and limonene, respectively.

Supplementary Note 4: Model Explanation for the Effect of Chiral Additives Near the Catalyst Surface.

4.1. Operational Definition and Spatial Distribution of Additives. We now consider a slightly more complex case of an achiral catalyst in the absence of a magnetic field (i.e., there is no global spin bias) but which exists in the vicinity of chiral additives embedded within its support (i.e., there is a local spin bias). An earlier generation of this model was proposed previously,[5] which we review and afterwards discuss refinements. At a high level, the influence of chiral additives is accounted for in a manner analogous to that of a global chiral bias, but the "domain of influence" of a single additive extends only locally over a limited number of sites. Every reaction intermediate in the lattice then experiences a unique bias that is dictated by the net influence of all nearby additives. We define this localized effect as a *local spin bias*, B_{LSB}. We assume that the local spin biases associated with these additives are randomly distributed both spatially and orientationally, at least when dipole-dipole interactions are neglected (*vide infra* for a discussion of this effect). To describe the spatial distribution of additives, we employ a procedure like that used to populate the lattice with reaction intermediates. That is, we directly superimpose a second, identical grid on top of the reaction intermediate lattice, and populate its sites with chiral additives by drawing from a binomial distribution with parameter Θ. This parameter describes the probability that a given site in the additive lattice is occupied and is analogous to the degree of additive loading within the catalyst support (i.e., assumed to be roughly proportional to the concentration of additives). Note that the lattice of additives is drawn to coincide with the lattice of reaction intermediates for the sake of convenience, so that the domains of influence centered on each additive do not partially overlap reaction intermediates on their boundaries. This choice greatly simplifies assignment of the local spin bias acting on each reaction intermediate.

4.2 Assignment of Orientation, Enantiomer, and Local Spin Bias to Each Additive. Each of these additives is assumed to cast a local, limited-range chiral bias B_{LSB} that otherwise acts in the same manner as the global bias B_{GSB} – that is, if a reaction intermediate is located within the domain of influence of an additive, the probability that it is spin-up is $0.5 \pm B_{\mathrm{LSB}}$ (subject to conditions on its orientation, as described below). The domain of influence is taken to be a square region centered on the additive's position. The orientation of the additive's chiral bias also matters: if an additive's enantiomeric form is S and it is oriented upward, its chiral bias will be $+B_{\mathrm{LSB}}$, but if its orientation is flipped, its chiral bias will be $-B_{\mathrm{LSB}}$. This behavior reflects the CISS-dependent change in spin polarization that manifests with molecular orientation.[6] Likewise, different enantiomers oriented in the same direction will produce opposing chiral biases. It is important to describe this behavior because when additive loading is high, their domains of influence may overlap, and enantiomeric or orientational disorder can cause chiral biases to cancel out. The enantiomer of each additive (R or S, corresponding to values of -1 or +1) is assigned by drawing from a binomial distribution with parameter ε, and the orientation of the additive (down or up, also corresponding to -1 and +1) is assigned by drawing from another binomial distribution with parameter α. (Here, we assume that S additives oriented upwards favor spin-up reaction intermediates, and inversion of either of these variables produces the opposite preference, i.e., upwards R additives and downwards S additives favor spin-down intermediates, but downwards R additives favor spin-up intermediates. This choice is arbitrary but does not influence the overall conclusions of the model. Formally, the chiral bias associated with a given additive located at row I and column

J in its grid is $B_{IJ} = |B_{\mathrm{LSB}}| E_{IJ} A_{IJ}$, where $E_{IJ}, A_{IJ} \in \{-1,1\}$ are the random variables drawn from distributions parameterized by ε and α, respectively.

4.3 Bias Combination Rule for Sites Influenced by Multiple Additives. When a reaction intermediate is influenced by multiple additives, the net bias is taken to be the arithmetic mean of their respective biases: $B_{ij} = \frac{1}{N}\sum_{I,J} B_{IJ}$, where the indices (I,J) run over all N additives whose domains of influence overlap site (i,j). In this way, we arrive at a model that replaces the global bias parameter, B_{GSB}, with four new ones: additive coverage parameter, Θ, additive bias strength, $|B_{\mathrm{LSB}}|$, additive enantiopurity, ε, and additive alignment factor, α. The net effect is that, in contrast with a global spin bias that acts uniformly over the entire catalyst patch, the spin bias in this case is different at every site in the lattice and itself varies probabilistically according to the spatial, orientational, and enantiomeric distributions of the chiral additives. Although modeling the behavior of a catalyst subject to a local spin bias is more complex than under a global bias, the effect of the local bias strength $|B_{\mathrm{LSB}}|$ on the triplet yield is qualitatively the same. That is, the triplet yield still follows a parabolic relationship with the bias strength; see Figure S10 for a comparison of enantiopure vs. racemic additives. The strength of this relationship is affected by the enantiopurity and alignment factors, illustrated by the fact that the maximum value on the curve corresponding to enantiopure additives does not reach the equivalent value in Figure 3 (i.e., the limiting case of uniform spin polarization), in the case of moderate spatial misalignment of the additives ($\alpha = 0.8$).

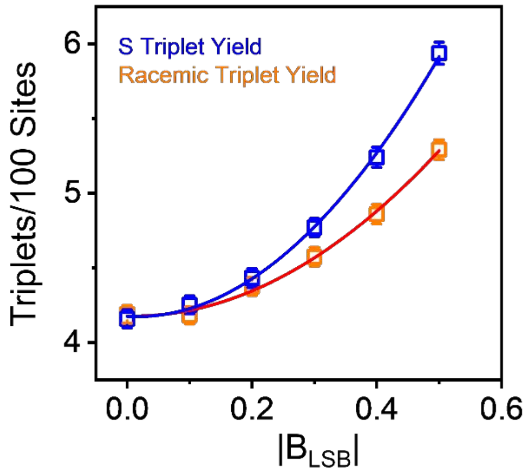

Figure S10. Simulated triplet yield of catalysts under the influence of enantiopure S (orange) and racemic (blue) additives of varying local spin bias strength $|B_{\mathrm{LSB}}|$ $\underline{\theta} = 0.5$, $P_{\uparrow\downarrow} = 0$, $P_{\uparrow\uparrow} = 0.1$, $\Theta = 0.1$, $\alpha = 0.8$, $D = 5$ sites, and $\varepsilon = 0.5$ for racemic additives and 1 for S additives. For simplicity, dipole-dipole interactions between additives are ignored in these simulations.

4.4 Dipole-Dipole Interactions Between Additives. The foregoing discussion assumes that dipole-dipole interactions between additives is negligible and that their orientation is determined solely by the parameter α. However, the electric field created by a dipole can cause nearby dipoles to adopt an orientation that opposes it, creating a misorientation effect in dense populations of additives with an initially consistent orientation.[7] (This behavior is analogous to the behavior of polar liquids with sub-unity Kirkwood g-factors,[8] signifying a tendency for local anti-parallel ordering of the constituent dipoles. The ordering behavior of polar liquids in

general can be dictated by other short-range interactions (e.g., induced dipole interactions, hydrogen bonds, and other steric effects), admitting a diverse range of possible motifs for which the Kirkwood factor may be greater (parallel ordering favored) or less (antiparallel ordering favored) than 1. Because the additive dipoles are less densely packed than they would be in a liquid, we retain only the interaction between permanent dipoles.) Thus, we expect that the advantage of enantiopure over racemic catalysts will deteriorate in cases of high loading, and that this effect will become more severe when the electric dipole moment of the additives is large. To account for this effect in the model, we take the additive distributions generated above as an initial condition, and perturb their orientations stochastically according to the total dipole-dipole interaction energy to drive the system toward a quasi-equilibrium configuration that respects Boltzmann statistics. To perform this calculation, we must compare the dipole-dipole interaction energy U_{DD} with the energy of the substrate-additive interaction implied by the orientation parameter α. We assume that α deviates from 0.5 because it is energetically preferable for the additives to be oriented in a way that favors spin-up reaction intermediates (assuming $\alpha > 0.5$; otherwise, the additives would favor spin-down reaction intermediates). This tendency towards coherent alignment may arise from interfacial electric fields near the catalyst surface, or steric constraints imposed during solidification of the catalyst-binder assembly. That is, the energy of an "upward" additive is less than that of a "downward" additive: $E_{\uparrow}^{0} < E_{\downarrow}^{0}$, all else being equal, and we assume this energy is the same for all additives. The probability that a given additive is upward-favoring is

$$\alpha = \frac{\exp\left(-\frac{E_{\uparrow}^{0}}{k_{\mathrm{B}}T}\right)}{\exp\left(-\frac{E_{\uparrow}^{0}}{k_{\mathrm{B}}T}\right) + \exp\left(-\frac{E_{\downarrow}^{0}}{k_{\mathrm{B}}T}\right)} = \frac{1}{1 + \exp\left(\frac{E_{\uparrow}^{0} - E_{\downarrow}^{0}}{k_{\mathrm{B}}T}\right)}$$

Without loss of generality, we may arbitrarily set the energy scale such that $E_{\uparrow}^{0} = 0$. Then, we the baseline orientational energy in terms of the orientation parameter can be expressed as:

$$E_{\downarrow}^{0} = k_{\mathrm{B}}T \ln \frac{\alpha}{1-\alpha}$$

(For $\alpha = 0.8$ and $T = 300$ K, this energy is approximately 36 meV.) The effect of dipole-dipole interactions is then dependent on how strong the interaction energy is in comparison with this value. The electrostatic interaction energy between two point dipoles of equal magnitude, μ, lying in a plane at distance r apart from one another is:

$$U_{\mathrm{DD}} = \pm \frac{1}{4\pi\epsilon_{\mathrm{r}}\epsilon_{0}} \frac{\mu^{2}}{r^{3}} ,$$

with the energy positive if the dipoles are parallel and negative if they are antiparallel (for simplicity, we assume that the dipole moments are oriented either upwards or downwards—i.e, no tilting), ϵ_{r} is the dielectric constant/relative permittivity of the intervening medium, and ϵ_{0} is the permittivity of free space. The net energy for a given additive at site (I,J) in its grid may then be expressed as the sum of the baseline orientational energy and the net interaction energies associated with all other additives in its vicinity:

$$U_{IJ} = \begin{cases} \sum_k U_{\mathrm{DD},k} \text{ ,oriented upward} \\ E_{\downarrow}^{0} + \sum_k U_{\mathrm{DD},k} \text{ ,oriented downward} \end{cases}$$

Due to the nature of this interaction, the orientation of any additive thus depends, in principle, on the orientation of all other additives present in the lattice; however, the importance of these additives falls off cubically with distance. For μ = 4.1 D, ϵ_r = 4, and r = 0.5 nm, parameters characteristic of closely packed camphorsulfonic acid molecules in the model, U_{DD} is approximately 21 meV when a single pair of additives is considered – i.e., on the same order of magnitude as the 36 meV alignment energy estimated above for α = 0.8. (For camphor and limonene (μ = 3.1 and 1.57 D), U_{DD} becomes 12 meV and 3.1 meV respectively, illustrating the reduction in the strength of this effect.) It is therefore reasonable to expect that the dipole-dipole interaction may not be especially important when additives are sparsely distributed (i.e., relatively large r), but dominant when additive concentration is high. Because the most probable state of each individual additive is not known a priori, we employ a variation of the Metropolis algorithm[4] to assign it such that the ensemble of additives represents the situation we would expect under thermal equilibrium. That is, we assign initial orientations of the additives according to the probability α, then the orientation is flipped stochastically until a quasi-equilibrium state is attained wherein the organizing force of the baseline orientational energy is in balance with the disorganizing force of the dipole-dipole interactions. This state is achieved by selecting an additive at random and calculating the energies associated with the "up" and "down" states defined above. For simplicity and computational efficiency, we calculate the net dipole-dipole interaction energy from additives present in a 5×5 square surrounding each additive, encompassing up to 7th-nearest neighbor interactions. If the orientation of the additive in question matches the high-energy state, it flips to the low-energy state; if it matches the low-energy state, it flips to the high-energy state with probability defined by the corresponding Boltzmann factor. This process is repeated 100 times per additive, giving the system an opportunity to converge to a quasi-equilibrium state. After the conclusion of the additive orientation assignment algorithm, the net chiral bias is determined and the singlet and triplet product pairings are calculated as described previously.

In Figure S11, we show several sample calculations of spin bias distributions that clarify the importance of including the dipole-dipole interactions, particularly in the dense coverage regime. When additives are sparsely distributed over the surface, the LSB distributions differ marginally in the cases whether dipole-dipole interactions are included or neglected, since the average distance between additives is too large. In this regime, LSBs from the additives display a coherent orientation when they are enantiopure, but are more evenly distributed between upward- and downward-favoring regions when they are racemic. By contrast, when additives are more dense, the effect of dipole-dipole interactions is strong enough that LSB distributions from racemic and enantiopure additive populations become less distinguishable. This comparison illustrates how, in aggregate, the triplet yield and specific activity of catalysts exhibit convergence of racemic and S-systems as additive loading increases.

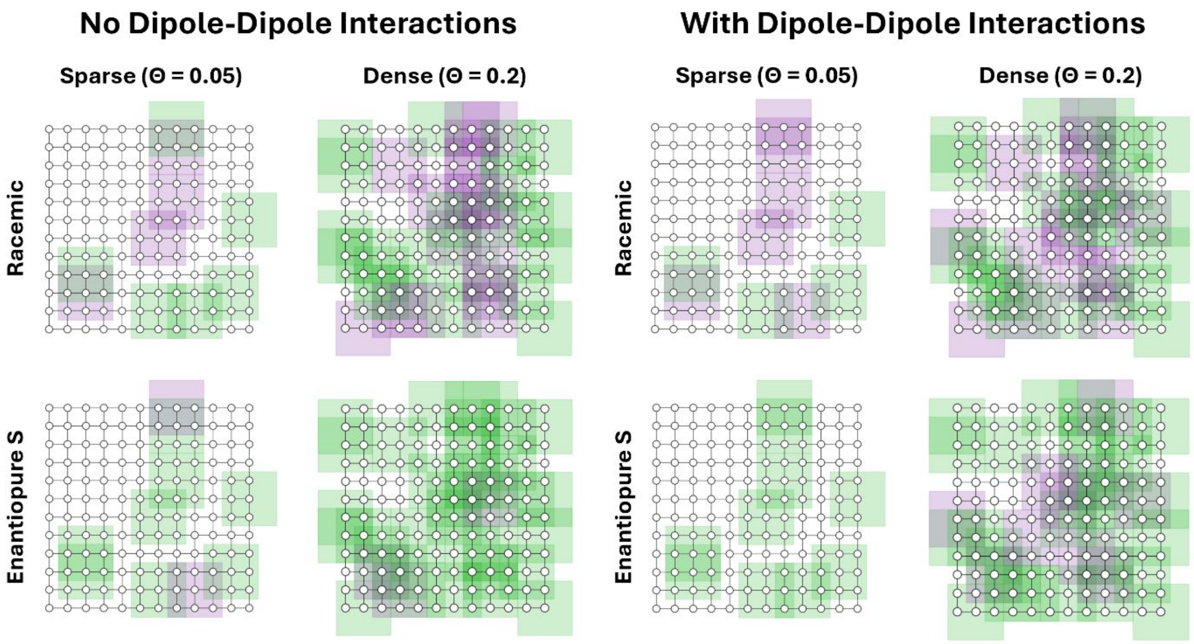

Figure S11. Sample calculations of local spin bias distributions due to chiral additives near the catalyst surface. Individual sites are shown as white circles; occupancy and spin states of the reaction intermediates are suppressed for clarity. Additive domains of influences are indicated by colored squares, with green ones denoting regions in which spin-up reaction intermediates are favored, and purple ones denoting downward-favoring spin biases. The left block of lattices is calculated without taking into account dipole-dipole interactions, while the right block includes interactions calculated using the following parameter set: temperature T = 300 K; lattice spacing d = 0.5 nm; additive dipole moment μ = 3.1 D; and dielectric constant ϵ_{r} = 4. In all calculations, the additive alignment factor α = 0.8; enantiopure additives are calculated using ε = 1 and racemic additives with ε = 0.5. 3×3 site domains of influence are employed for ease of visualization.

4.5 Resolution of Distinct Effects of Additive Dipole Moment. Besides the simulations presented in the main text in Figure 4, we performed additional calculations with the dipole-dipole interaction mechanism disabled, in order to better illuminate the effects of the individual mechanisms by which the additive dipole moment affects OER activity. These simulations are presented in Figure S12, and we employ the same parameter set as used in Figure 4, except that we investigate all combinations of additive bias strength $|B_{\mathrm{LSB}}| \in \{0.3, 0.4, 0.5\}$ and domain of influence side length $D \in \{3, 5, 7\}$ to further discriminate between the effects of these parameters. Note that the results in the panels on the figure diagonal correspond directly to those in Figure 4 with the dipole-dipole interactions suppressed. It is immediately clear that, without the dipole-dipole interactions, the triplet yield of catalysts with enantiopure additives remains elevated above that of catalysts with racemic additives, and in fact levels off, particularly when the additive bias strength is high. This behavior disagrees with the experimentally determined specific activity (Figure 4a-c) and therefore supports the hypothesis that dipole-dipole interactions are important for describing the effect of chiral additives on OER. Another important effect is that the maximum triplet yield increases strongly with additive bias strength, but only weakly with domain size, and this trend holds regardless of whether the additives are racemic or enantiopure. In addition, this behavior accounts for the increase in SA with additive dipole moment at the optimal additive concentration, since each individual additive has a more potent effect on the catalyst. Put another way, the maximum triplet yield is attained when the catalyst surface is covered by enough additives that most

reaction intermediates are affected by a chiral bias, but not so much that overlap between conflicting additives leads to destructive interference. Thus, the maximum triplet yield is largely controlled by the additive bias strength (stronger bias leads to higher triplet yields), while the additive loading at which it occurs is mostly determined by the domain size ("larger" additives lead to this maximum being reached at lower concentrations). Collectively, these simulations show that the additive dipole moment has a complicated effect on OER activity: on one hand, the stronger spin bias and larger domain size are beneficial, but dipole-dipole interactions limit the amount of additives that can be usefully introduced before destructive interference hampers the beneficial effects.

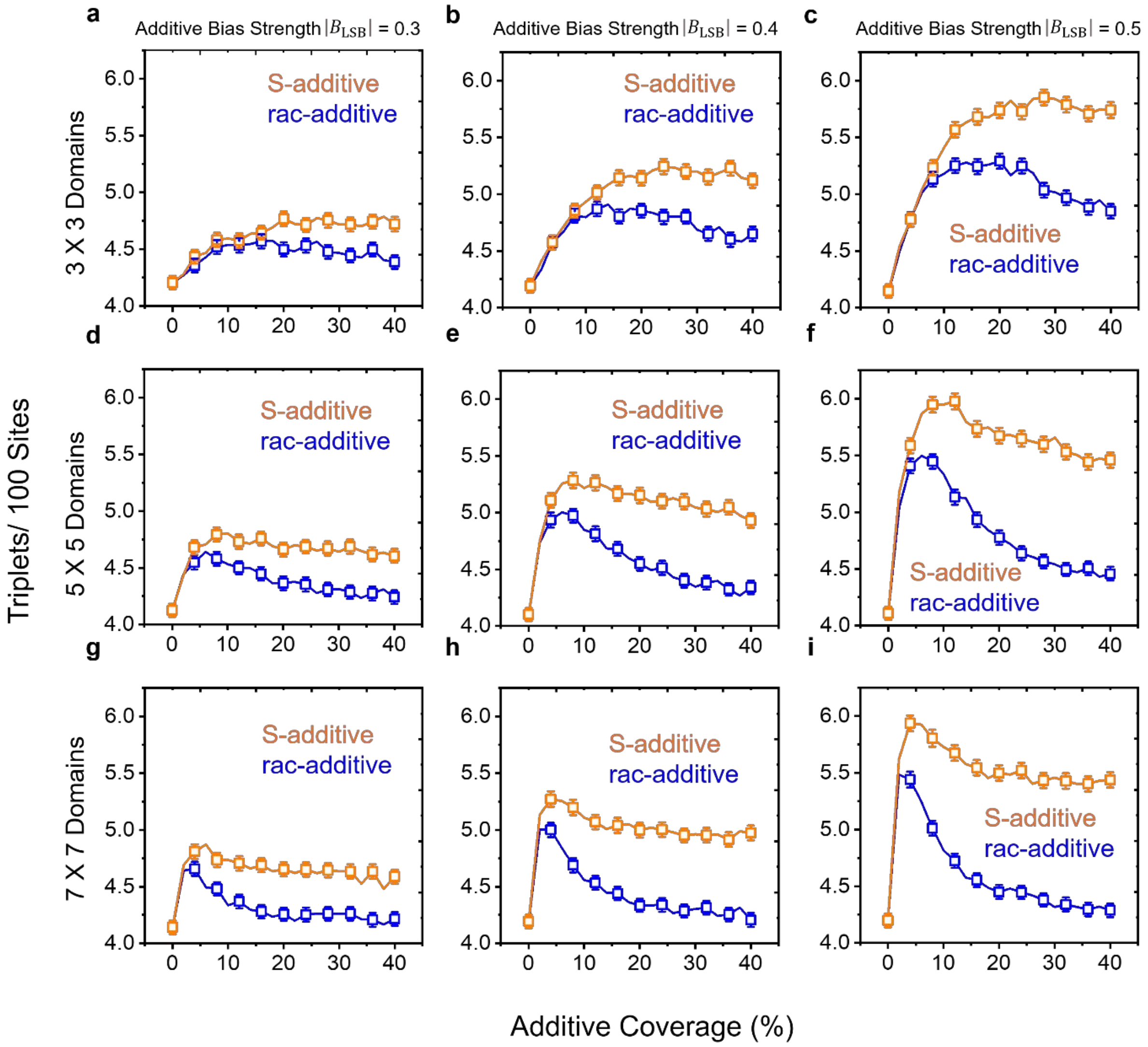

Figure S12. Monte Carlo simulations of the effects of chiral additives on OER efficacy, considering both racemic (ε = 0.5, blue traces) and enantiopure (ε = 1, orange traces) additives, with dipole-dipole interactions deliberately suppressed. All parameters are fixed except for additive bias strength and domain of influence, with reaction intermediate coverage θ = 0.5, triplet pairing probability $P_{\uparrow\uparrow}$ = 0.1, singlet pairing probability $P_{\uparrow\downarrow}$ = 0, and additive alignment factor α = 0.8. The top, middle, and bottom rows correspond to simulations with D = 3, 5, and 7-site wide domains of influence; the left, middle, and right columns correspond to simulations with additive bias strength $|B_{\mathrm{LSB}}|$ of 0.3, 0.4, and 0.5. Each data point represents the mean of 1000 simulations; error bars represent 95% confidence intervals about each mean.

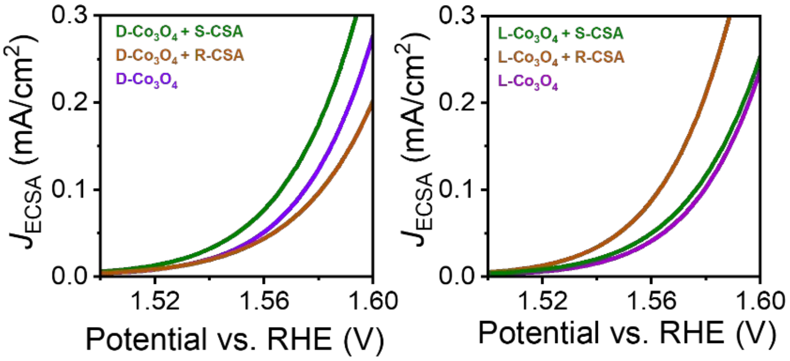

Figure S13. Linear sweep voltammograms of D-Co_3O_4 (left panel) with S-CSA (green) and R-CSA (brown), and without (Violet) any additives to the catalyst matrix. Right panel shows LSVs of L-Co_3O_4 with S-CSA (green) and R-CSA (brown), and without (purple) any additives to the catalyst matrix. Each curve represents the average of three independently prepared electrodes.

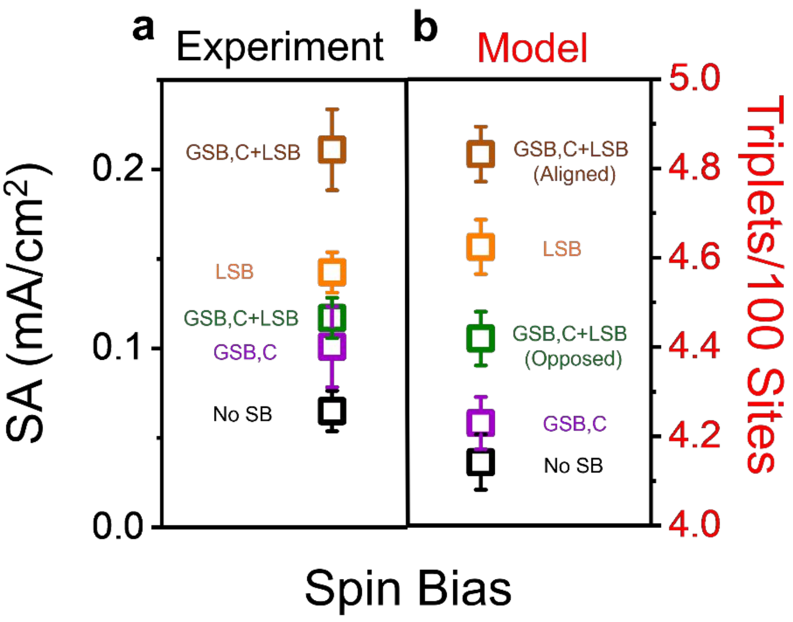

Figure S14. (**a**) presents experimental measurements of specific activity at 350mV overpotential for catalysts under the influence of the different local spin bias of chiral additives and global spin bias for L-Co_3O_4 (purple), L-Co_3O_4 + S-CSA (green), L-Co_3O_4 + R-CSA (brown). The black and orange symbols correspond to rac-Co_3O_4 and rac-Co_3O_4 + S-CSA respectively. Each experimental data point represents measurements from three independently prepared electrodes, and the error bars indicate their standard deviation. (**b**) shows Monte Carlo simulations of triplet yield using the same parameter set as Figure 6 (D-Co_3O_4), except using a weaker global spin bias $|B_{\mathrm{GSB,C}}| = \pm\ 0.1$. In contrast to the D-$Co_3O_4$, the intrinsic spin bias of the catalyst is so weak that the presence of chiral additives is beneficial regardless of whether the global and local spin biases are aligned or opposed (although the former case produces the highest performance, as expected). Each data point in the model represents the mean of 1000 simulations, and the error bars represent 95% confidence intervals about this mean.

Supplementary Note 5: Combination of Spin Biases from Chiral and Magnetic Sources.

The introduction of the magnetic field as another source of spin polarization raises the question of whether we should continue to average the biases, as done for purely chiral systems, or seek an alternative treatment. We propose that, due to the distinct physical origins of chiral and magnetic biases, the latter course is appropriate. As noted in the main text, we average the biases due to multiple chiral additives and, if applicable, a global chiral bias because this method preserves the ability to penalize systems in regions where they exhibit heterochirality, and retain favorable behavior only where the chiral biases are mutually coherent. This scheme is in harmony with the observation that spin-polarized electrons are blocked when homochirality is interrupted,[9] which may severely reduce their polarization (or that of chemical populations affected by them). By contrast, a magnetic field should be expected to modulate the polarization produced by a chiral system in a continuous manner commensurate with its strength. We have already noted in the main text and in Supplementary Note 2 that, assuming the ensemble of intermediates behaves as a two-state paramagnet with individual magnetic moments m, the effective bias produced by a magnetic field H alone is

$$B_{\mathrm{GSB,M}} = \frac{1}{2}\tanh\left(\frac{mH}{k_{\mathrm{B}}T}\right) .$$

We may combine this bias with the net chiral bias from the catalyst and any additives present by using an analogous equation to determine the effective magnetic field H_χ due to chiral sources, adding it to the applied field, and recalculating the bias. That is, for the chiral sources, $B_\chi = \frac{1}{2}\tanh\left(\frac{mH_\chi}{k_{\mathrm{B}}T}\right)$. (At this stage, we are agnostic as to whether the chiral bias B_χ arises from additives, a chiral catalyst, or a combination of both.) To calculate the effective field, it is useful to work with the spin-up probability corresponding to the bias, $P_\uparrow = \frac{1}{2} + B_\chi$:

$$P_\uparrow = \frac{1}{2}\left(1 + \tanh\left(\frac{mH_\chi}{k_{\mathrm{B}}T}\right)\right)$$

Expanding the hyperbolic tangent,

$$P_\uparrow = \frac{1}{2}\left(\frac{\exp\left(\frac{mH_\chi}{k_{\mathrm{B}}T}\right) + \exp\left(-\frac{mH_\chi}{k_{\mathrm{B}}T}\right)}{\exp\left(\frac{mH_\chi}{k_{\mathrm{B}}T}\right) + \exp\left(-\frac{mH_\chi}{k_{\mathrm{B}}T}\right)} + \frac{\exp\left(\frac{mH_\chi}{k_{\mathrm{B}}T}\right) - \exp\left(-\frac{mH_\chi}{k_{\mathrm{B}}T}\right)}{\exp\left(\frac{mH_\chi}{k_{\mathrm{B}}T}\right) + \exp\left(-\frac{mH_\chi}{k_{\mathrm{B}}T}\right)}\right)$$

Simplifying,

$$P_\uparrow = \frac{1}{2}\left(\frac{2\exp\left(\frac{mH_\chi}{k_{\mathrm{B}}T}\right)}{\exp\left(\frac{mH_\chi}{k_{\mathrm{B}}T}\right) + \exp\left(-\frac{mH_\chi}{k_{\mathrm{B}}T}\right)}\right) = \frac{1}{1 + \exp\left(-\frac{2mH_\chi}{k_{\mathrm{B}}T}\right)}$$

Replacing our original definition of the bias,

$$\frac{1}{2} + B_\chi = \frac{1}{1 + \exp\left(-\frac{2mH_\chi}{k_{\mathrm{B}}T}\right)}$$

Rearranging,

$$\exp\left(-\frac{2mH_\chi}{k_\mathrm{B}T}\right) = \frac{1}{\frac{1}{2}+B_\chi} - 1 = \frac{\frac{1}{2}-B_\chi}{\frac{1}{2}+B_\chi}$$

Taking the logarithm of both sides yields the final expression for the effective magnetic field corresponding to the net chiral bias:

$$H_\chi = -\frac{k_\mathrm{B}T}{2m}\ln\left(\frac{\frac{1}{2}-B_\chi}{\frac{1}{2}+B_\chi}\right)$$

Note that while this derivation is performed for the chiral bias, it is completely general and applies equally well to the relationship between an actual magnetic field and the spin bias it produces. This equivalence allows us to construct a net bias from N_b separate sources in terms of their effective magnetic fields H_k:

$$\frac{1}{2} + B_\mathrm{net} = \frac{1}{1+\exp\left(-\frac{2m}{k_\mathrm{B}T}\sum_{k=1}^{N_\mathrm{b}} H_k\right)}$$

Inserting the above expression for each magnetic field component in terms of the bias B_i it produces:

$$B_\mathrm{net} = \frac{1}{1+\exp\left(-\frac{2m}{k_\mathrm{B}T}\sum_{k=1}^{N_\mathrm{b}} - \frac{k_\mathrm{B}T}{2m}\ln\left(\frac{\frac{1}{2}-B_k}{\frac{1}{2}+B_k}\right)\right)} - \frac{1}{2}$$

It is interesting to note here the useful result that the dependence of the net bias on the physical parameters is eliminated from the final result. Simplifying,

$$B_\mathrm{net} = \frac{1}{1+\exp\left(\sum_{k=1}^{N_\mathrm{b}} \ln\left(\frac{\frac{1}{2}-B_k}{\frac{1}{2}+B_k}\right)\right)} - \frac{1}{2}$$

Exploiting the fact that the output of an exponential function whose argument can be expressed as a sum of variables is equal to the product of exponentials of those individual variables – that is, $\exp\sum_k x_k = \prod_k \exp x_k$,

$$B_\mathrm{net} = \frac{1}{1+\prod_{k=1}^{N_\mathrm{b}} \exp\left(\ln\left(\frac{\frac{1}{2}-B_k}{\frac{1}{2}+B_k}\right)\right)} - \frac{1}{2}$$

We thereby arrive at the general result:

$$B_{\text{net}} = \frac{1}{1+\prod_{k=1}^{N_b}\left(\frac{\frac{1}{2}-B_k}{\frac{1}{2}+B_k}\right)} - \frac{1}{2}$$

For the case of interest wherein we investigate the combination of the magnetic bias B_{m} and net chiral bias B_{χ},

$$B_{\text{net}} = \frac{1}{1+\left(\frac{\frac{1}{2}-B_{\chi}}{\frac{1}{2}+B_{\chi}}\right)\left(\frac{\frac{1}{2}-B_{\text{m}}}{\frac{1}{2}+B_{\text{m}}}\right)} - \frac{1}{2}$$

We can confirm that when one of these biases (say, B_{m}) is zero, the net bias correctly reduces to the other one:

$$B_{\text{net}} = \frac{1}{1+\left(\frac{\frac{1}{2}-B_{\chi}}{\frac{1}{2}+B_{\chi}}\right)\left(\frac{\frac{1}{2}-0}{\frac{1}{2}+0}\right)} - \frac{1}{2} = \frac{1}{1+\left(\frac{\frac{1}{2}-B_{\chi}}{\frac{1}{2}+B_{\chi}}\right)} - \frac{1}{2} = \frac{\frac{1}{2}+B_{\chi}}{\frac{1}{2}+B_{\chi}+\frac{1}{2}-B_{\chi}} - \frac{1}{2}$$

$$B_{\text{net}} = \frac{\frac{1}{2}+B_{\chi}}{1} - \frac{1}{2}$$

$$B_{\text{net}} = B_{\chi}$$

Note also that the above expression for the combination of two biases can be condensed into a more compact form:

$$B_{\text{net}} = \frac{B_{\chi}+B_{\text{m}}}{1+4B_{\chi}B_{\text{m}}}$$

The theoretical framework of the magnetization of a two-state paramagnet thereby furnishes us with a useful approach that we may apply to the combination of biases. We show the results of simulations of a catalyst subject to the joint influence of chiral additives and a magnetic field in Figure S15 (i.e., $B_{\chi} = B_{\text{LSB,net}}$ and $B_{\text{m}} = B_{\text{GSB}}$). Comparison of the constant-GSB curves against the equivalent ones plotted in Figure 5 for a chiral catalyst demonstrates several important differences. While the overall behavior is broadly similar, the magnetic field is considerably more capable of overcoming misalignment with the prevailing bias of the additives than chirality of the catalyst. Notably, the regime of synergy, in which opposed biases can still generate larger triplet yields than either bias acting alone, extends over a much larger region of the parameter space. When the biases are aligned, their effects remain cooperative, if not necessarily optimal, regardless of additive coverage or field strength. This behavior – especially the expanded regime of synergy – explains the experimental observation in Figure 7 that, although performance is most improved when a magnetic bias is aligned with that of the additives, even an opposed bias can also contribute beneficially.

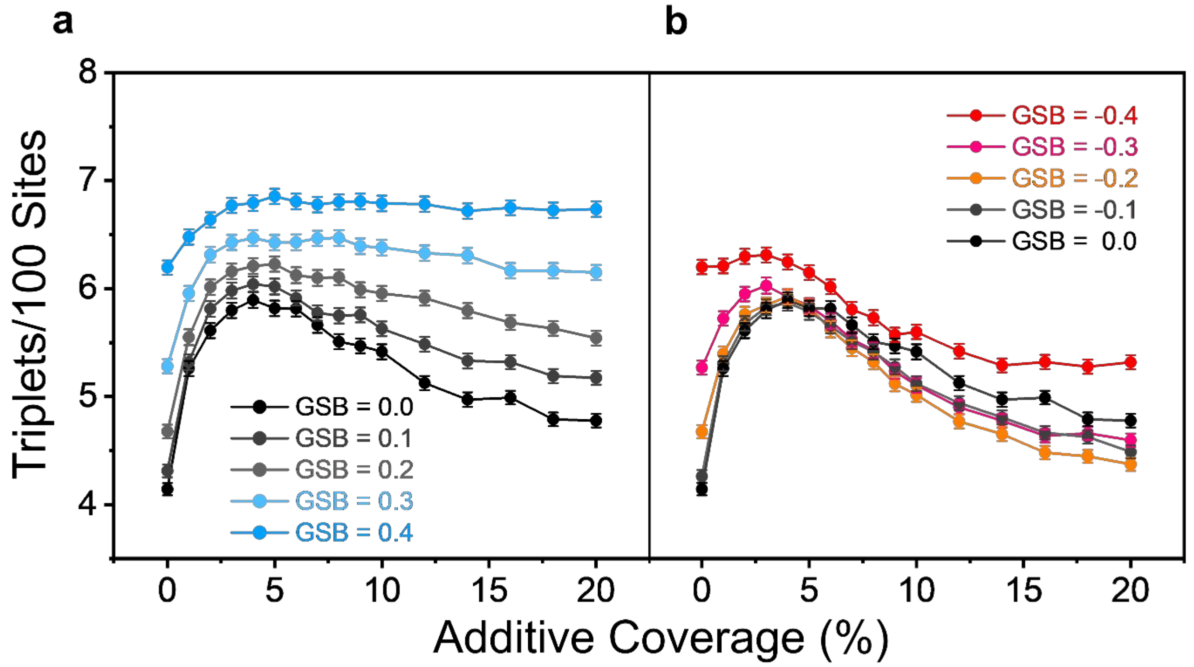

Figure S15. Monte Carlo simulations of triplet yield for a catalyst under the joint influence of chiral additives and a magnetic field associated with global spin bias B_{GSB}, as a function of chiral additive coverage parameter Θ. As in Figure 5, we retain additive parameters characteristic of enantiopure CSA—i.e., bias strength $|B_{\text{LSB}}| = 0.5$, 7-site wide additive domain of influence, dipole moment $\mu = 4.1$ D, enantiopurity factor $\varepsilon = 1$, and alignment factor $\alpha = 0.8$—as well as reaction intermediate coverage parameter $\theta = 0.5$, fix triplet and singlet reaction rate parameters $P_{\uparrow\uparrow} = 0.1$ and $P_{\uparrow\downarrow} = 0$, temperature $T = 300$ K, dielectric constant $\epsilon_r = 4$, and lattice site spacing $d = 0.5$ nm. (**a**) corresponds to cases in which the prevailing bias of the additives is aligned to that of the magnetic field; (**b**) corresponds to cases in which they are opposed. Note that the "regime of synergy," the region in which the combined effect of an opposed GSB and LSB improves triplet yield more than either one acting alone, extends for additive coverages $\Theta \in [0\%, 4\%]$ and $B_{\text{GSB}} \in [-0.4, 0]$. Each data point represents the mean of 1000 simulations; error bars represent 95% confidence intervals about the mean.

Supplementary Note 6: Activation of Magnetic Catalysts

Potentiostatic bulk electrolysis was carried out to change the oxidation state of the rac-, L-, and D-Co_3O_4 nanomaterials, as they initially exhibited no magnetic dependence in their electrochemical behavior. Figure S16a, b and c show LSV curves for the rac-, L-, and D-Co_3O_4 nanomaterials measured at OER potential conditions both in the presence (solid lines) and absence (dashed lines) of an external magnetic field. After applying a potential of 2 V vs. RHE, following a previously reported method for creating CoO_x,[10] the nanomaterials began to display magnetic properties, as shown in Figure S16d, e & f. This emergence of a magnetic response in the materials has previously been attributed to the formation of more paramagnetic Co(IV) centers upon oxidation.[10,11,12] Notably, the chirality effect remained intact even after bulk electrolysis, as demonstrated by the higher current response of L-, or D-Co_3O_4 compared to its racemic counterpart.

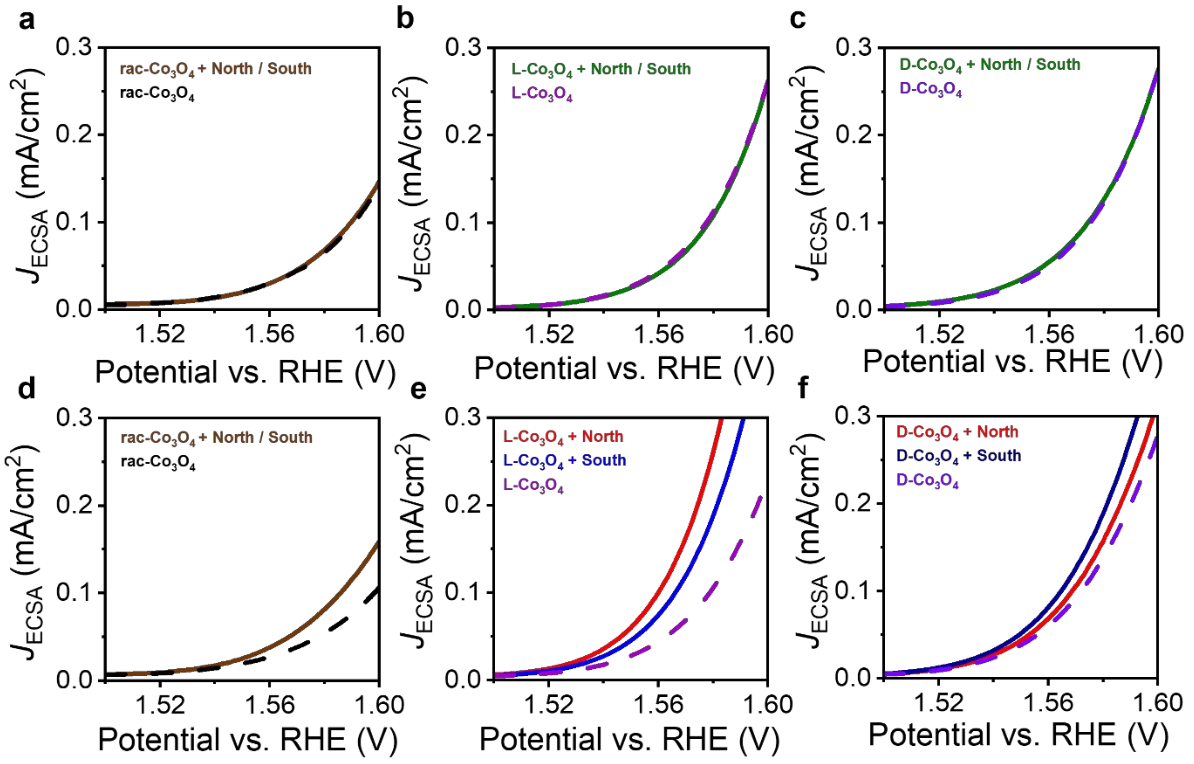

Figure S16. Linear sweep voltammograms of rac-, L- & D-Co_3O_4 nanomaterials before (**a, b, c**) and after (**d, e, f**) bulk electrolysis, in the presence (solid line) and absence (dashed line) of a magnetic field. Each curve represents the average of three independently prepared electrodes.

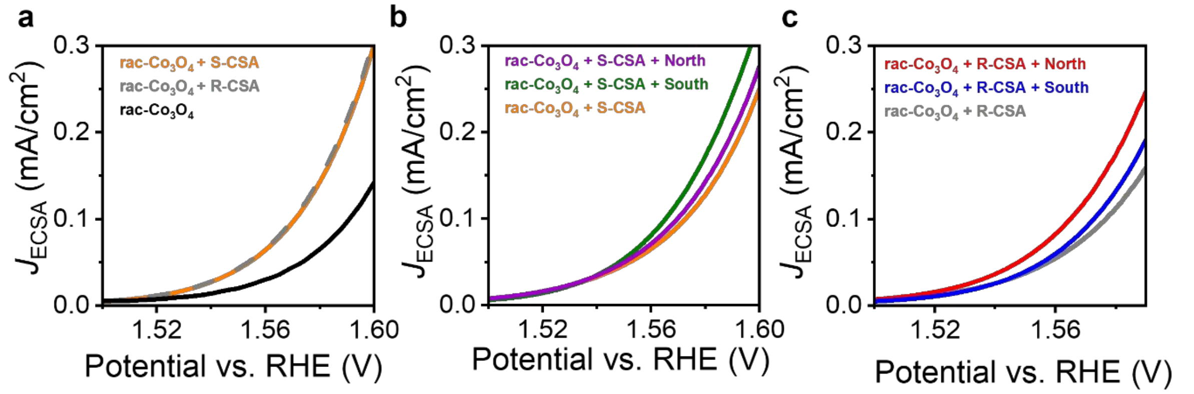

Figure S17. Linear sweep voltammograms of (**a**) rac-Co_3O_4 with S-CSA additives (green), R-CSA additives (gray), and without additives (Violet). Panel (**b**) & (**c**) shows LSVs of rac-Co_3O_4 with S-CSA & R-CSA in the presence of North and South magnetic fields, respectively. Each curve represents the average of three independently prepared electrodes.

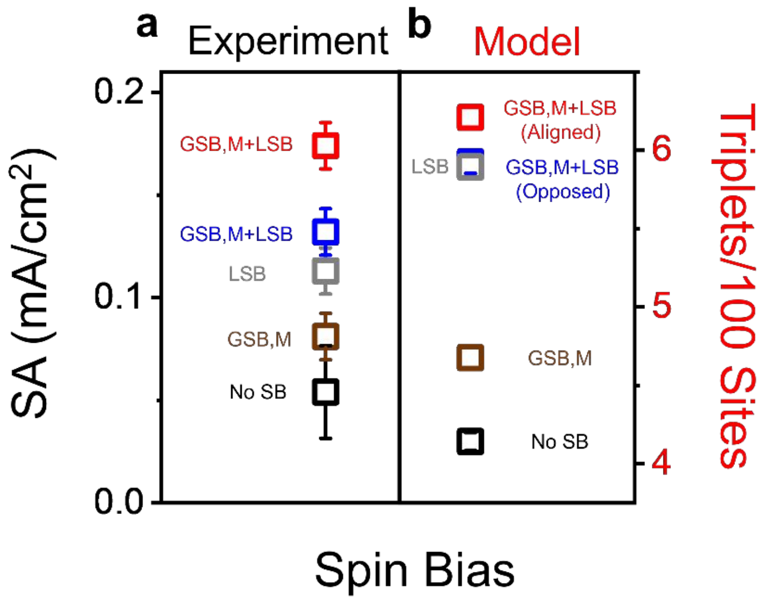

Figure S18. (**a**) presents experimental measurements of specific activity at 350mV for rac-Co_3O_4 (black), in the presence of a North or South magnetic field (brown), or R-CSA chiral additives (gray). The SA values for rac-Co_3O_4 combined with R-CSA and South field (North field) are represented in blue (red). Each experimental data point represents measurements from three independently prepared electrodes, and the error bars indicate their standard deviation. (**b**) shows Monte Carlo simulations of triplet yield using parameter combinations that reproduce the ordering of OER efficiency metrics for that of an applied magnetic field as a weak global spin bias that is locally overridden by the spin bias of any additives present. Each data point in the model represents the mean of 1000 simulations, and the error bars represent 95% confidence intervals about this mean; however, these may appear visually compressed due to the larger size of the data markers.

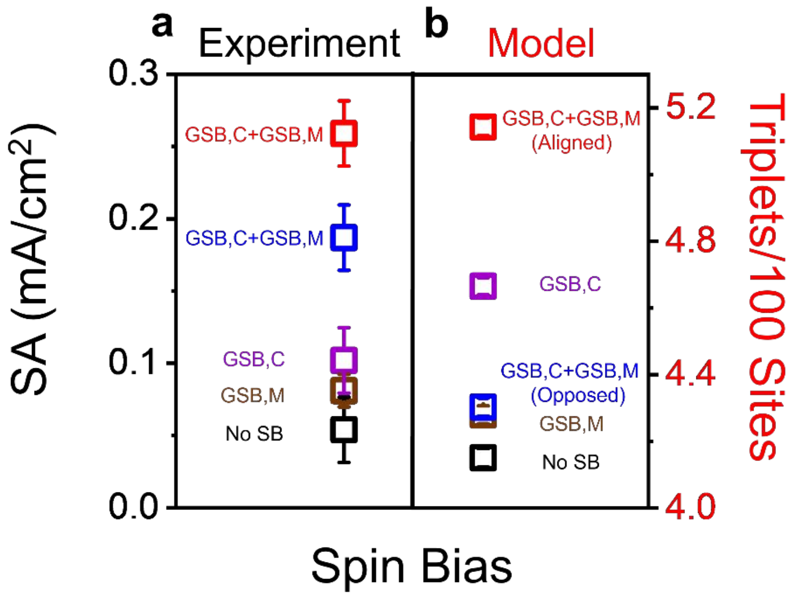

Figure S19. (**a**) shows experimentally measured specific activity at 350mV overpotential for L-Co_3O_4 (purple), L-Co_3O_4 in the presence of a North field (red), or South field (blue). The brown and black symbols show SA of rac-Co_3O_4 in the presence and absence of an applied magnetic field. Each

experimental data point represents measurements from three independently prepared electrodes, and the error bars indicate their standard deviation. (**b**) shows Monte Carlo simulations of triplet yield using parameter combinations that reproduce the ordering of OER efficiency metrics for that of an applied magnetic field as a weak global spin bias that is locally overridden by the spin bias of any additives present. Each data point in the model represents the mean of 1000 simulations, and the error bars represent 95% confidence intervals about this mean.

List of Variables

Symbol	Definition	Context
A, A_{IJ}	Dipole moment orientation of a chiral additive located in row I and column J in its lattice; symmetric binary variable with values +1 (upwards) or -1 (downwards)	Model variable
B_{GSB}	Global spin bias of arbitrary origin	Model parameter
$B_{\mathrm{GSB,C}}$	Global spin bias from catalyst chirality	Model parameter
$B_{\mathrm{GSB,M}}$	Global spin bias from an applied magnetic field	Model parameter
B_{IJ}	Local spin bias produced by a chiral additive located in row I and column J in its lattice	Model variable
$\lvert B_{\mathrm{LSB}} \rvert$	Unsigned strength of the local spin bias from chiral additives	Model parameter
$B_{\mathrm{GSB,net}}$	Net global spin bias affecting all reaction intermediates equally, due to the joint effect of a magnetic field and inherent chirality of a catalyst	Model variable
$B_{\mathrm{LSB,net}}$	Net spin bias acting on a particular reaction intermediate due to the joint effect of several overlapping additives	Model variable
B_{net}	Net spin bias acting on a particular reaction intermediate due to the joint effect of global and local spin biases	Model variable
B_{χ}	Net spin bias acting on a particular reaction intermediate due to the joint effect of global and local spin biases from chiral sources only	Model variable
d	Distance between adjacent sites in the lattice	Model parameter
D	Edge length of chiral additives' domain of influence in number of lattice sites	Model parameter
E, E_{IJ}	Enantiomeric type of the chiral additive located in row I and column J in its lattice: symmetric binary variable with values +1 (S) or -1 (R)	Model variable
$E_{\downarrow}^{0}$	Orientational energy associated with a chiral additive with dipole moment pointing downwards, neglecting dipole-dipole interactions	Model parameter
$E_{\uparrow}^{0}$	Orientational energy associated with a chiral additive with dipole moment pointing upwards, neglecting dipole-dipole interactions	Model parameter
ECSA	Electrochemically accessible surface area	Experimental measurement
H	Applied magnetic field	Experimental measurement
H_{χ}	Effective magnetic field associated with a spin bias from a chiral source	Model variable
i	Row index for sites in the reaction intermediate lattice	Model index
I	Row index for sites in the additive lattice	Model index
j	Column index for sites in the reaction intermediate lattice	Model index
J	Column index for sites in the additive lattice	Model index
k	Dummy index	Model index
k_{B}	Boltzmann's constant	Physical constant
m	Magnetic dipole moment of a reaction intermediate	Model parameter
M	Magnetization of an ensemble of reaction intermediates	Model variable

M_∞	Saturation magnetization of an ensemble of reaction intermediates	Model variable
n	Side length of a simulated patch of catalyst surface	Model parameter
N	Number of additives influencing the spin state of a specific reaction intermediate	Model variable
$N_\uparrow$	Number of upward-oriented reaction intermediates in a given lattice	Model variable
$N_\downarrow$	Number of downward-oriented reaction intermediates in a given lattice	Model variable
N_T	Total number of reaction intermediates in a given lattice	Model parameter
o_{ij}	Occupancy of a site in the reaction intermediate lattice at row i and column j: asymmetric binary random variable with value 0 (vacant) or 1 (occupied)	Model variable
O_{IJ}	Occupancy of a site in the additive lattice at row I and column J: asymmetric binary random variable with value 0 (vacant) or 1 (occupied)	Model variable
$P_\uparrow$	Probability that a specific reaction intermediate is spin-up	Model variable
$P_\downarrow$	Probability that a specific reaction intermediate is spin-down	Model variable
$P_{\uparrow\downarrow}$	Probability that spin-opposed reaction intermediates form a singlet product given the opportunity	Model parameter
$P_{\uparrow\uparrow}$	Probability that spin-aligned reaction intermediates form a triplet product given the opportunity	Model parameter
r	Distance between two additives located at arbitrary points	Model variable
S_A	Orientation of a chiral additive's local spin bias, dictated by both its electric dipole moment orientation and enantiomeric type: symmetric binary random variable with values +1 (favors spin-up intermediates) or -1 (favors spin down intermediates)	Model variable
SA	Specific activity	Experimental measurement
s_{ij}	Spin state of a reaction intermediate located at row i and column j in its lattice: symmetric binary random variable with values +1 (spin up) or -1 (spin down)	Model variable
T	Temperature	Model parameter
U_DD	Interaction energy between two electric dipoles	Model variable
U_{IJ}	Net energy associated with a chiral additive's orientation, incorporating both dipole-dipole interactions from other additives as well as contributions from other sources associated with the alignment factor α	Model variable
Y_T	Triplet yield: number of triplet reaction products formed per 100 sites on a patch of catalyst surface	Model variable
α	Probability that a given chiral additive has its electric dipole moment oriented upwards	Model parameter
ε	Probability that a given chiral additive is the S enantiomer (rather than R)	Model parameter
ϵ_0	Permittivity of free space	Physical constant
ϵ_r	Dielectric constant/relative permittivity	Model parameter
θ	Occupation probability of sites in the reaction intermediate lattice	Model parameter
Θ	Occupation probability of sites in the chiral additive lattice	Model parameter
μ, EDM	Electric dipole moment of chiral additives	Model parameter
ρ	Weight factor used to calculate the net effect of combined global and local spin biases	Model parameter
σ_{ij}	Joint spin-occupancy state of a reaction intermediate located at row i and column j in its lattice: ternary random variable with values +1 (occupied, spin-up), -1 (occupied, spin-down), or 0 (vacant)	Model variable

References

1 Vadakkayil, A.; Clever, C.; Kunzler, K. N.; Tan, S.; Bloom, B. P.; Waldeck, D. H. Chiral electrocatalysts eclipse water splitting metrics through spin control. *Nat. Commun*. **2023**, *14*, 1067.

2 Yeom, J.; Santos, U. S.; Chekini, M.; Cha, M.; de Moura, A. F.; Kotov, N. A. Chiromagnetic nanoparticles and gels. *Science*. **2018,** *359*, 309–314.

3 McCrory, C. C. L.; Jung, S.; Peters, J. C.; Jaramillo, T. F. Benchmarking Heterogeneous Electrocatalysts for the Oxygen Evolution Reaction. *J. Am. Chem. Soc.* **2013**, *135, 45*, 16977–16987.

4 Schroeder, D. V. *An Introduction to Thermal Physics*, **2000**, Addison Wesley Longman, San Francisco

5 Vadakkayil, A.; Dunlap-Shohl, W. A.; Joy, M.; Bloom, B. P.; Waldeck, D. H. Improved Catalyst Performance for the Oxygen Evolution Reaction under a Chiral Bias. *ACS Catal.* **2024**, *14*, 23, 17303–17309

6 Bloom, B. P.; Paltiel, Y.; Naaman, R.; Waldeck, D. H. Chiral Induced Spin Selectivity. *Chem. Rev.* **2024**, *124* (3), 1950–1991.

7 Lu, Z.; Manias, E.; Macdonald, D. D.; Lanagan, M. Dielectric Relaxation in Dimethyl Sulfoxide/Water Mixtures Studied by Microwave Dielectric Relaxation Spectroscopy. *J. Phys. Chem. A* **2009**, *113, 44*, 12207–12214.

8 Kirkwood, J. G. The Dielectric Polarization of Polar Liquids. *J. Chem. Phys.* **1939**, *7*, 911-919.

9 Wei, J.; Bloom, B. P.; Dunlap-Shohl, W. A.; Clever, C. B.; Rivas, J. E.; Waldeck, D. H. Examining the Effects of Homochirality for Electron Transfer in Protein Assemblies. *J. Phys. Chem. B* **2023**, 127, 29, 6462–6469.

10 Ghosh, S.; Bloom, B. P.; Lu, Y.; Lamont, D.; Waldeck, D. H. Increasing the Efficiency of Water Splitting through Spin Polarization Using Cobalt Oxide Thin Film Catalysts. *J. Phys. Chem. C.* **2020**, *124*, 22610–22618.

11 Hunt, C.; Zhang, Z.; Ocean, K.; Jansonius, R. P.; Abbas, M.; Dvorak, D. J.; Kurimoto, A.; Lees, E. W.; Ghosh, S.; Turkiewicz, A.; Garcés Pineda, F. A.; Fork, D. K.; Berlinguette, C. P. Quantification of the Effect of an External Magnetic Field on Water Oxidation with Cobalt Oxide Anodes. *J. Am. Chem. Soc.* **2022**, *144*, 2, 733–739

12 Casella, I. G.; Gatta, M. Study of the electrochemical deposition and properties of cobalt oxide species in citrate alkaline solutions. *J. Electroanal. Chem*. **2002**, *534*,31−38.
